# A systematic review of national interventions and policies to optimize antibiotic use in healthcare settings in England

**DOI:** 10.1093/jac/dkae061

**Published:** 2024-03-20

**Authors:** Rebecca Knowles, Clare Chandler, Stephen O’Neill, Mike Sharland, Nicholas Mays

**Affiliations:** Department of Health Services Research and Policy, London School of Hygiene and Tropical Medicine, London, UK; Department of Global Health and Development, London School of Hygiene and Tropical Medicine, London, UK; Department of Health Services Research and Policy, London School of Hygiene and Tropical Medicine, London, UK; Centre for Neonatal and Paediatric Infection, Institute for Infection and Immunity, St George’s, University of London, London, UK; Paediatric Infectious Diseases Department, St George’s University Hospitals NHS Foundation Trust, London, UK; Department of Health Services Research and Policy, London School of Hygiene and Tropical Medicine, London, UK

## Abstract

**Objectives:**

To identify and assess the effectiveness of national antibiotic optimization interventions in primary and secondary care in England (2013–2022).

**Methods:**

A systematic scoping review was conducted. Literature databases (Embase and Medline) were used to identify interventions and evaluations. Reports included the UK AMR Strategy (2013–2018), National Action Plan (2019–2024) and English Surveillance Programme for Antimicrobial Utilisation and Resistance (ESPAUR) reports (2014–2022). The design, focus and quality of evaluations and the interventions’ effectiveness were extracted.

**Findings:**

Four hundred and seventy-seven peer-reviewed studies and 13 reports were screened. One hundred and three studies were included for review, identifying 109 interventions in eight categories: policy and commissioning (*n* = 9); classifications (*n* = 1); guidance and toolkits (*n* = 22); monitoring and feedback (*n* = 17); professional engagement and training (*n* = 19); prescriber tools (*n* = 12); public awareness (*n* = 17); workforce and governance (*n* = 12).

Most interventions lack high-quality effectiveness evidence. Evaluations mainly focused on clinical, microbiological or antibiotic use outcomes, or intervention implementation, often assessing how interventions were perceived to affect behaviour. Only 16 interventions had studies that quantified effects on prescribing, of which six reported reductions. The largest reduction was reported with structural-level interventions and attributed to a policy and commissioning intervention (primary care financial incentives). Behavioural interventions (guidance and toolkits) reported the greatest impact in hospitals.

**Conclusions:**

Many interventions have targeted antibiotic use, each pulling different levers across the health system simultaneously. On the basis of these studies, structural-level interventions may have the greatest impact. Collectively, the combination of interventions may explain England’s decline in prescribing but direct evidence of causality is unavailable.

## Introduction

Antimicrobial resistance (AMR) led to an estimated 1.27 million deaths from drug-resistant infections globally in 2019, and is partly related to inappropriate antibiotic use in healthcare.^1,2^ It is considered a top 10 global health threat by WHO.^3^ An international commitment to AMR was signalled through a resolution at the 2015 World Health Assembly, which saw the launch of a Global Action Plan (GAP) and a commitment for UN member states to develop their own National Action Plans (NAPs) to address AMR in prioritized areas of action, including the optimization of antimicrobial use.^4,5^

The UK has been a significant leader in AMR policy and action. The first UK AMR Strategy was published in 2000, subsequently followed by an AMR action plan in 2011, a 2013–2018 Strategy and its successor the 2019–2024 NAP (of which the last two align with the GAP).^6,7^ These plans set the policy direction for addressing AMR, outlining key targets and interventions.^8,9^ England is successfully reaching these targets, including reducing human antimicrobial use by 15% by 2024, and is seeing a sustained reduction in antibiotic prescribing that could indicate it is effectively implementing strategies to optimize antibiotic consumption.^10^

While the NAP sets the strategy, it is the role of the health system to develop and implement antimicrobial stewardship (AMS) activities to achieve targets. In England, the National Health Service (NHS) is responsible for most healthcare, delivered in primary care by general practitioner (GP) practices and in secondary care by hospitals organized into NHS trusts. Clinical Commissioning Groups (CCGs) were responsible for planning local services and allocating two-thirds of the NHS budget until 2021, and have since been replaced by Integrated Care Systems.^11,12^ The UK Health Security Agency (UKHSA) (previously Public Health England) oversees public health, while the Department of Health and Social Care is the government department leading national policy.

Interventions for optimizing antibiotic use act at all points along the prescribing pathway, including addressing professional and public knowledge. The term ‘intervention’ is defined broadly in this case, referring to policies, guidelines, activities and technologies intended to improve health outcomes. They are ‘restrictive’ if they use rules to reduce opportunities for specific behaviours (e.g. limiting certain antibiotics) or ‘enabling’ if they increase opportunities or capabilities for change (e.g. education or awareness-raising campaigns).^13^ It is intrinsically difficult to evaluate many of these interventions because they are often not amenable to experimental methods (such as randomized controlled trials, RCTs), and they tend to be introduced alongside others simultaneously.

Although key strategies are described in the NAP, there is yet to be an assessment of the wide range of different interventions implemented in England, and the extent to which they have been successful. This review therefore aimed to identify the types of national intervention used in England between 2013 and 2022 to optimize antibiotic use in primary and secondary care and to assess their effectiveness using peer-reviewed evaluations and grey literature reports produced by UK central government departments and their agencies.

## Methods

Since the evidence to be reviewed covered different interventions, study designs, outcomes and settings, the review followed Arksey and O’Malley’s framework for scoping reviews with some modifications suggested by Levac *et al.*^14–16^

The research question was: what is known from existing literature about interventions used to optimize antibiotic prescribing and their effectiveness in England? The sub-questions were: what types of intervention have been implemented; how have interventions been evaluated and what types of intervention have been most effective?

**Figure 1.**
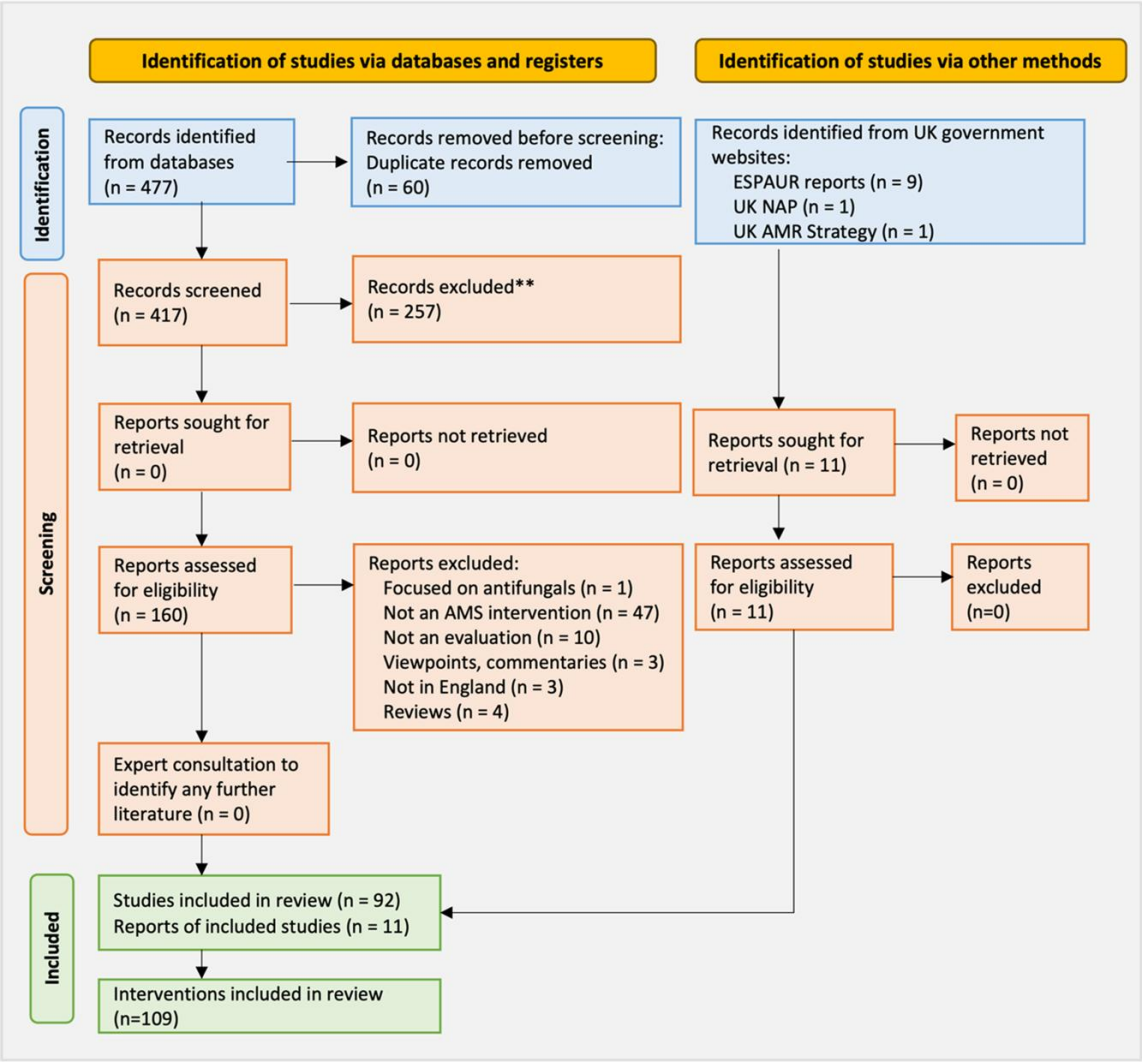
PRISMA diagram outlining the process for identifying, screening and including studies for the scoping review.^17^ This figure appears in colour in the online version of *JAC* and in black and white in the print version of *JAC*.

Websites (UK Government, Department of Health and Social Care, UKHSA) and literature databases (Embase and Medline) were used to identify interventions and evaluations (Figure 1, Box 1). Reports identified were the UK AMR Strategy (2013–2018), NAP (2019–2024) and English Surveillance Programme for Antimicrobial Utilisation and Resistance (ESPAUR) reports (2014–2022).^8,18,19^

Box 1. **Embase and Medline search terms**

- (‘antibiotic use’ OR ‘antibiotic utilisation’ OR ‘antibiotic prescribing’ OR ‘antibiotic prescription’ OR ‘antibiotic stewardship’ OR ‘antimicrobial use’ OR ‘antimicrobial stewardship’)
  AND
- (evaluat* OR ‘policy evaluation’ OR ‘programme evaluation’ OR ‘process evaluation’ OR ‘outcome evaluation’ OR ‘impact evaluation’ OR ‘context evaluation’ OR ‘effect’)
  AND
- (‘United Kingdom’ or UK or England or ‘Great Britain’)
  NOT
- (farm* or cat or dog or animal or vet)

Titles and abstracts were screened. Full text screening of all potentially relevant studies was conducted using the inclusion criteria:

- Intervention aims to optimize antibiotic use
- Study conducted in England or intervention focused on all regions of the UK
- Intervention implemented between 2013 and 2023 (aligning with the development and implementation of the two NAPs)
- Human healthcare settings only. Studies or interventions targeting both primary and secondary care settings are included.

Exclusion criteria:

- Interventions outside the UK or only affecting Scotland, Wales or Northern Ireland
- Interventions in animals and agriculture
- Antimicrobial surveillance, infection prevention control (including hand hygiene) or vaccines
- Reviews, commentaries or expert opinions.

The data extracted included: intervention name and type, author, year, study design, evaluation methods and key results. Interventions and evaluations were grouped according to the intervention type, evaluation focus and study design (influenced by research assessing the quality of AMS evaluations).^20^ Studies that had a quantifiable antibiotic use (referring to the consumption of antibiotics by a patient) or prescribing outcome (the drugs prescribed by a clinician or dispensed by a pharmacy) were identified. Interventions were deemed to have higher quality evidence of effectiveness if this was obtained through an RCT or an appropriate quasi-experimental design. The themes that emerged from the included studies were used to identify and categorize interventions, informed also by other studies that defined categories of interventions globally and in other countries.^15,21,22^

Experts were consulted [regional AMS leads and representatives from the UK Government’s Advisory Committee on Antimicrobial Prescribing, Resistance and Healthcare Associated Infection (APRHAI)] after the analysis to identify whether the results aligned with their experiences (and if not, in what way).

## Results

### Types of intervention identified

**Figure 2.**
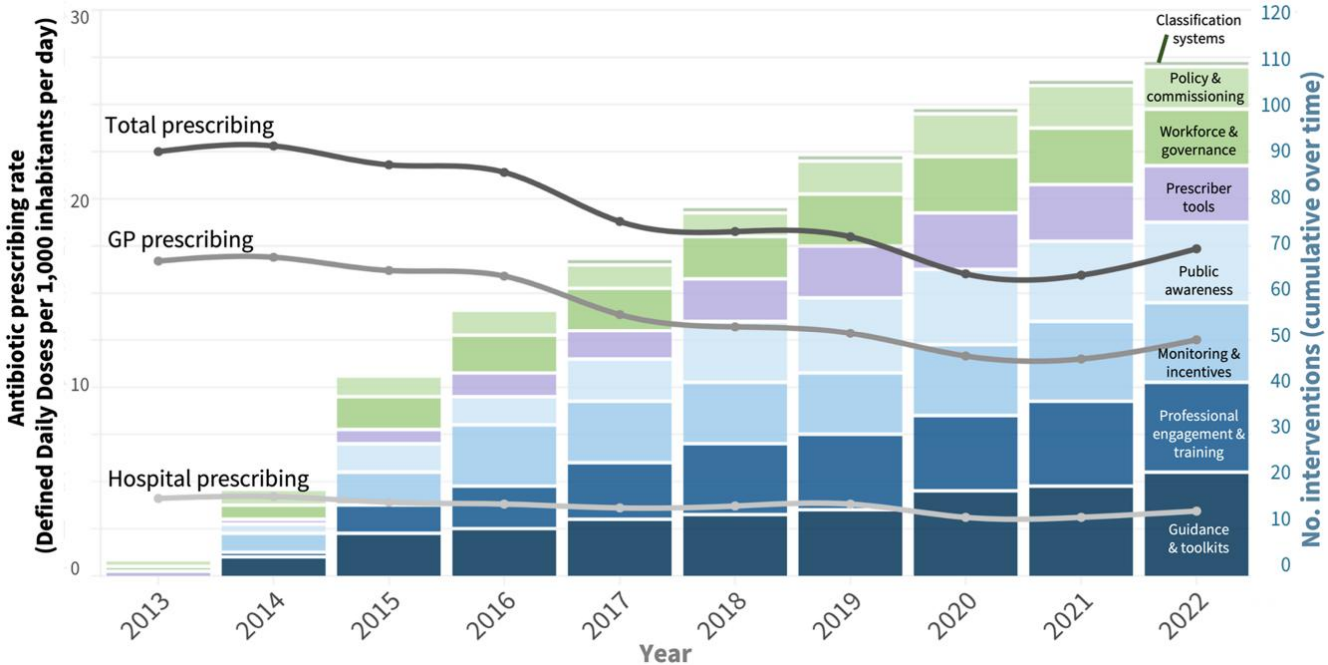
Cumulative number of interventions targeting antimicrobial use and prescribing rates in England between 2013 and 2022. Prescription data from ESPAUR reports (2014–2023). This figure appears in colour in the online version of *JAC* and in black and white in the print version of *JAC*.

**Figure 3.**
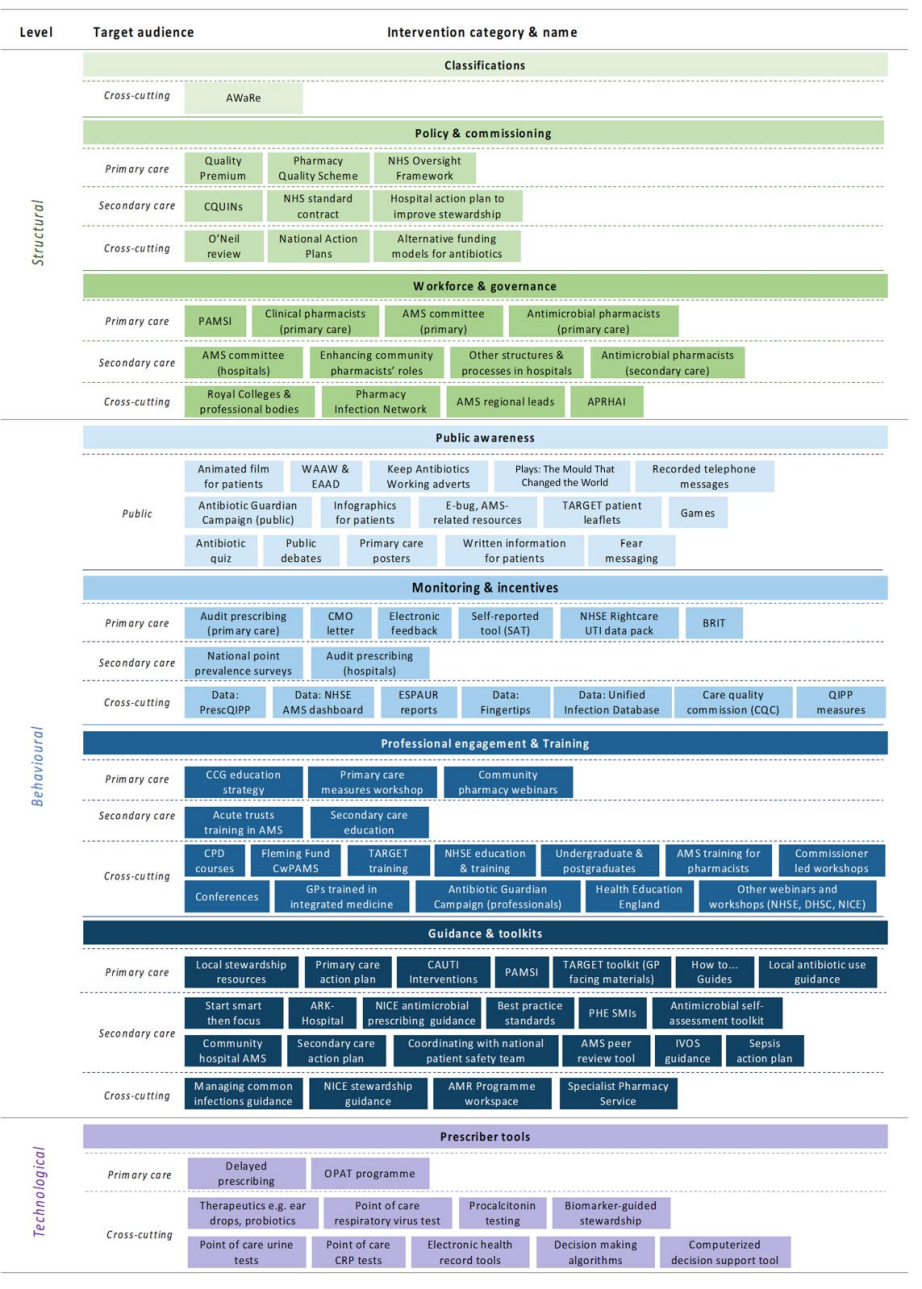
List of interventions by type and target audience. APRHAI, Advisory Committee on Antimicrobial Prescribing, Resistance and Healthcare Associated Infection; PAMSI, Community Pharmacy Antimicrobial Stewardship Intervention; AMS, antimicrobial stewardship; CPD, Continuing Professional Development; Fleming Fund CwPAMS, Commonwealth Partnerships for Antimicrobial Stewardship; OPAT, outpatient parenteral antimicrobial therapy; WAAW, World Antibiotic Awareness Week; EAAD, European Antibiotic Awareness Day; BRIT, Building Rapid Interventions to Reduce Antibiotic Resistance; QIPP, quality, innovation, productivity and prevention; CAUTI, community acquired urinary tract infection; IVOS, intravenous to oral switch. This figure appears in colour in the online version of *JAC* and in black and white in the print version of *JAC*.

Between 2013 and 2022, 109 antibiotic interventions were identified in England from 103 reports and publications. There has been a sustained increase in the number of interventions reported since the UK AMR Strategy in 2013, which coincided with a decrease in antibiotic use (Figures 2 and 3).

Seventy-five interventions used a behavioural approach to influence prescribing (22 guidance and toolkits; 17 monitoring and feedback; 19 professional engagement and 17 public awareness interventions). Twenty-two deployed structural approaches (nine were policy and commissioning interventions, one involved classification systems and 12 affected workforce and governance structures). Twelve used a technological approach to influence prescribing (all of which were prescriber tools like diagnostic tests) (Table 1, Figures 2 and 3).

**Table 1.** Definitions of intervention categories

Level	Category of intervention	Definition
Structural	Classifications	Interventions based on the categorization of elements relevant to the antibiotic use system, such as drug classification systems such as AWaRe
	Policy and commissioning	Interventions involving the planning, prioritizing and purchasing of services to achieve specific goals, such as financial incentive mechanisms
	Workforce and governance	Changes to organizational structures and job roles that are involved in antibiotic prescribing, such as embedding clinical pharmacists in primary care
Behavioural	Guidance and toolkits	Resources used by healthcare professionals and AMS committees to recommend and inform them of appropriate care and services, such as guidelines for specific indications
	Monitoring and feedback	Interventions based on the collection, use and communication of data to inform clinicians about patterns in prescribing, such as data platforms and dashboards
	Professional engagement and training	Educational and awareness-raising interventions that target professionals (e.g. pharmacists, clinicians, medical students), such as conferences, courses and campaign schemes
	Public awareness	Strategies to engage and educate people who are not experts or professionals in AMR, such as TV adverts
Technological	Prescriber tools	Devices, strategies and tests used by clinicians at the moment of prescription that can affect decisions about prescriptions, such as diagnostic tests

### Effectiveness of different types of intervention

**Figure 4.**
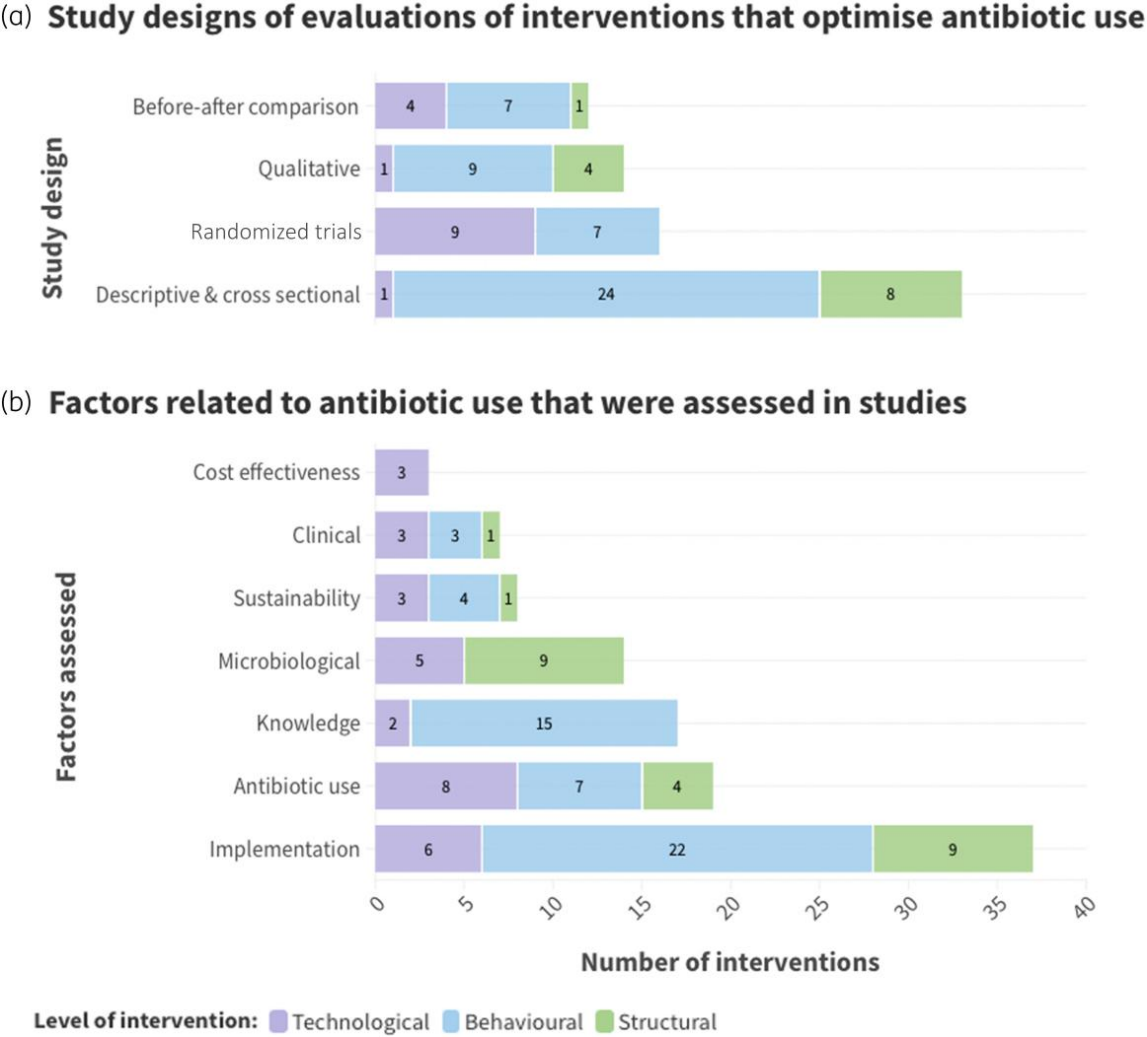
Factors assessed in studies evaluating antibiotic use interventions in England. Percentage reflects the proportion of interventions with an evaluation on each outcome for behavioural, structural and technological categories of intervention. This figure appears in colour in the online version of *JAC* and in black and white in the print version of *JAC*.Factors assessed:
Implementation includes: quantitative outcomes (e.g. adherence to guidelines, use of intervention materials, perceptions of interventions from surveys) as well as qualitative studies (e.g. perceptions of interventions from interviews, barriers and facilitators of implementing interventions) and process evaluationAntibiotic use outcomes include: number of antibiotics prescribed (e.g. per GP practice, per month, per STAR-PU, by AWaRe category), any antibiotic prescribed (yes/no), defined daily doses (e.g. total, per bed days), patient-reported antibiotic use, duration of antibiotic use, achieving antibiotic use targetsKnowledge outcomes include: self-reported change in knowledgeMicrobiological outcomes include: blood cultures, number of isolates tested against antibiotic during antimicrobial susceptibility testing, resistance to at least one antibioticSustainability: if evaluations covered at least 12 monthsClinical outcomes include: infection incidence, symptom or disease severity, symptom or infection duration, length of hospital stay, reattendance to primary care, diagnostic resultsCost effectiveness: if evaluated.

**Figure 5.**
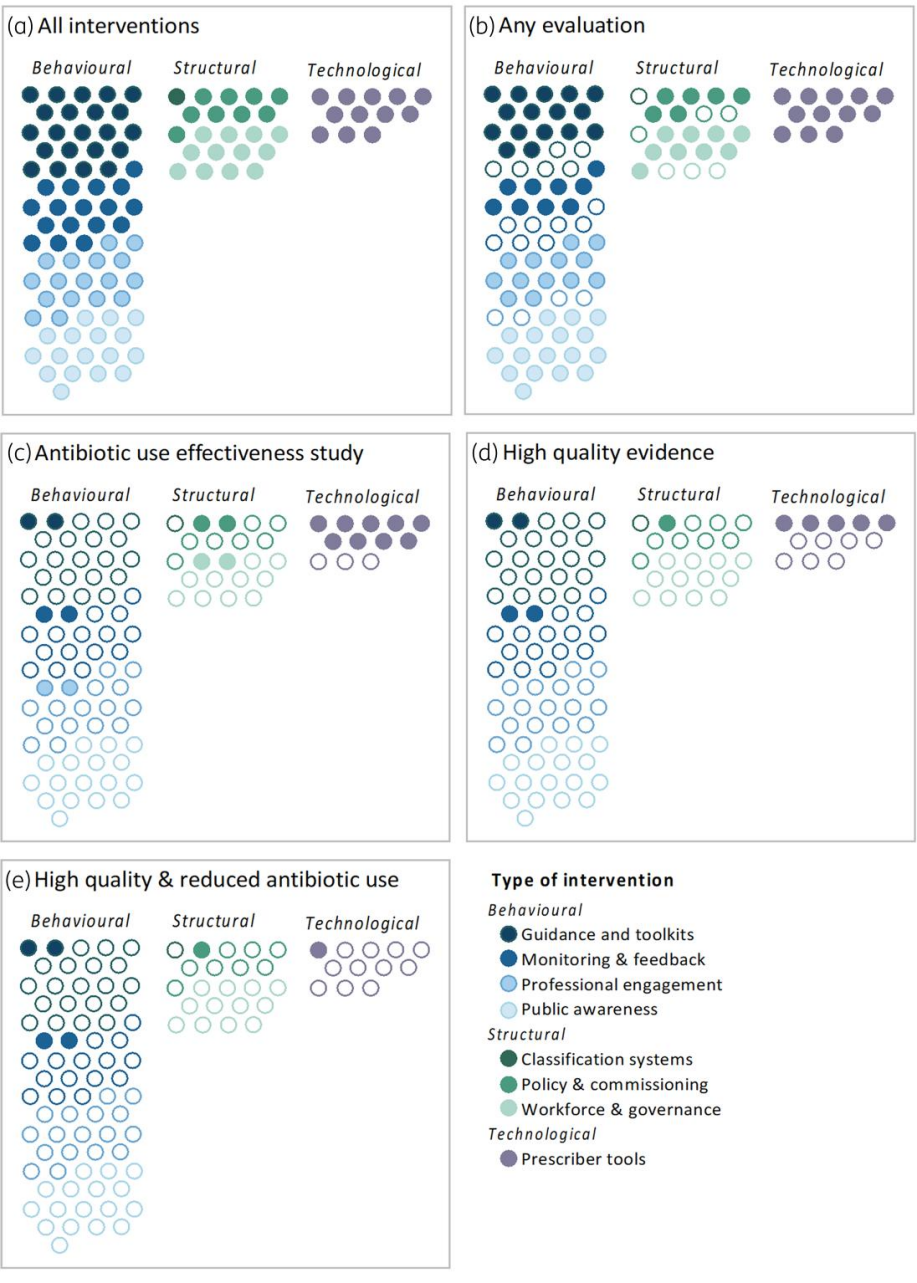
Evidence for the effectiveness of antibiotic use interventions in England. ABU, antibiotic use. This figure appears in colour in the online version of *JAC* and in black and white in the print version of *JAC*.Each circle represents an intervention. Circles are coloured in where they meet the criteria for that intervention:
Any evaluation if any study (qualitative or quantitative) has been conducted. Categories are ordered alphabeticallyABU effectiveness study if a quantitative evaluation with an antibiotic use outcome has been conductedHigh-quality evidence if an RCT or appropriate quasi-experimental design study has been conducted, with an antibiotic use outcomeHigh-quality and reduced ABU if there is evidence from an RCT or a study using an appropriate quasi-experimental design that the intervention has significantly reduced antibiotic prescribing.

A wide range of study designs (qualitative, cross-sectional, before–after comparisons, randomized trials) were used to evaluate 109 interventions. While 19 interventions had higher quality studies (defined as either an RCT or a study using an appropriate quasi-experimental design such as a controlled interrupted time series analysis), many studies were retrospective, conducted after an intervention ended or were cross-sectional surveys capturing perspectives of interventions at a single timepoint (Figures 4 and 5). One structural intervention had been evaluated by higher quality studies, compared to two behavioural interventions (both were guidance and toolkits) and five technological interventions (mainly diagnostics). Sustainability was assessed for nine interventions, with evaluations over more than 12 months.

Evaluations mainly focused on clinical, microbiological, antibiotic use, prescribing or implementation outcomes [Figure 4; Tables S1 and S2 (available as Supplementary data at *JAC* Online)]. Most studies assessed how interventions were perceived to affect individual behaviour, using self-reported indicators of implementation or success rather than reporting changes in prescribing. Of the potential metrics that can be used as outcomes to assess the impact of AMS activities, five interventions were evaluated with microbiological outcomes, eight reported clinical outcomes and 19 reported a quantifiable effect on antibiotic use or prescribing. Most evaluations that assessed an impact on prescribing were prescriber tools that influence prescribing through a technological approach (*n* = 9, out of 12 interventions). There were fewer structural (*n* = 4, out of 22) and behavioural (*n* = 6, out of 75) interventions evaluated with such outcomes (Figures 4 and 5, Table S1).

Six interventions reported a significant effect on antibiotic use, of which two were behavioural interventions, one was at the structural level and one used a technological approach. The structural-level intervention (the Quality Premium, a primary care financial incentive) showed the largest impact reporting a 57% reduction in prescribing over 72 months in 6882 GP practices.^23^ The behavioural approaches were guidance and toolkit interventions in secondary care (the Antibiotic Review Kit-hospital toolkit, which saw total antibiotic use reduced 4.8% per year, in 39 hospitals over 14 months) and providing feedback to clinicians (the Chief Medical Officer’s (CMO) letter, which reduced antibiotic prescribing 3% over 5 months).^24,25^ Two technological level interventions reduced antibiotic use, with a computerized decision support tool reducing prescribing by 12% in 79 GP practices over 12 months, and a C-reactive protein (CRP) diagnostic reducing the number of chronic obstructive pulmonary disease patients prescribed antibiotics by 22% (653 patients over 6 months).^26,27^

Overall, most interventions deployed a behavioural approach, with fewer higher quality studies of their impact on antibiotic use. Fewer interventions were implemented at the structural level, but these showed the greatest impact. Interventions using a technological approach were least common, although most of these had been evaluated through a higher quality study and two showed small effects. Each intervention category is described next. Some interventions overlap across categories, and many depend on or enable interventions in another category.

#### Policy and commissioning (structural)

Commissioning organizations and prescribers have been encouraged to implement AMS activities through target-based financial incentives. These interventions have been evaluated using relatively robust interrupted time series analyses (allowing before–after comparisons of prescribing), and qualitatively to understand how they had an effect from the perspectives of implementing teams.

In England, examples include the *Commissioning for Quality and Innovation* (CQUIN) in hospitals, the *Quality Premium* (*QP*) in primary care and the Pharmacy Quality Scheme in community pharmacies. In primary care the antibiotic QP was introduced in 2015 (later becoming the ‘NHS Oversight Framework’) to provide financial rewards to CCGs if they met certain indicators, e.g. a 1% reduction in total primary care prescribing and for broad spectrum antibiotics to be <10% of use. CCGs also developed their own financial incentives based on local priorities and needs.^28,29^

Evaluations of financial incentives showed they are an effective approach for reducing prescribing in England. The QP reporting the largest reduction in primary care prescribing out of all interventions assessed in this study, although this effect was not maintained longer term (Table S3).^23,28–30^ Qualitative studies revealed that financial incentives could help reduce prescribing by helping local teams prioritize AMS, but also demonstrated concerns that funding available was small and not clear how it fed back into healthcare teams.^31,32^

#### Classification systems (structural)

Classifications can form the basis of indicators assessing progress, such as the *AWaRe* (Access Watch and Reserve) system. AWaRe was introduced into WHO’s Model Essential Medicine’s List in 2017 which categorizes antibiotics into three groups: Access (first- and second-choice antibiotics for treating common infections), Watch (antibiotics with a higher resistance potential) and Reserve (last resort treatments).^33^ It underlies prescribing guidance in the AWaRe Book.^34^ England was the first country to adapt AWaRe in its NAP in 2019.^8,35^

AWaRe has been a key component of national targets and the NHS Standard Contract, and could affect prescribing in many ways as it becomes more embedded in data systems, targets, education and guidance.^36^ Such targets help focussing attention on AMR across the health care sector, and between 2017 and 2021, Access and Watch antibiotic consumption decreased by 4.2% and 7.1% respectively, while Reserve use increased 3.0%.^10^

#### Guidance and toolkits (behavioural)

Guidelines and toolkits influence antibiotic use, particularly in primary care where prescribing often relies on clinical judgement of symptoms rather than a diagnostic test.^37^ These have mainly been evaluated with cross-sectional studies using surveys to gather feedback about an intervention at one timepoint, although stronger evidence from RCTs is available for some interventions.

NICE and UKHSA created evidence-based clinical infection *antimicrobial prescribing guidelines* (2014) for primary and secondary care, as well as *guidance on stewardship* interventions (2015), which are updated regularly following changing resistance patterns or evidence.^38,39^ Other guidance included resources checklists for prescribers and audit templates to support commissioners. For example, the *Treat Antibiotics Responsibly Guidance, Education and Tools (TARGET) toolkit* was first created by UKHSA with the Royal College of General Practitioners in 2009, and has been actively promoted by almost all CCGs.^40,41^ In secondary care, the *Start Smart Then Focus (SSTF)* toolkit has been used since 2011, outlining evidence-based guidance for prescribers and AMS committees while the *Antibiotic Review Kit for hospitals (ARK-hospital)* supports clinical reviews (implemented 2017–2019).

Studies have measured the uptake of guidance such as TARGET and SSTF through surveys, and deployed qualitative methods to understand user perspectives.^18,42–47^ There was widespread knowledge of the resources available to support prescribers, but only an RCT of ARK-Hospital in 39 hospitals assessed the impact on prescribing, showing it was associated with a gradual reduction in antibiotic use and mortality over 14 months.^48^ Guidance was more likely to be effective if implemented as a package alongside training.^44,47^

#### Monitoring and feedback (behavioural)

National, regional, primary and secondary care antibiotic use has been monitored and reported to prescribers through data platforms since 2015, audits and social norms tactics (influencing behaviour without the force of law by using the rules that are understood by members of a community).^49,50^ Data platforms have been evaluated qualitatively, whereas social norms interventions have stronger evidence of their effect on prescribing from RCTs.

Data platforms included UKHSA’s *Fingertips* platform (launched 2016) and NHS-funded *PrescQIPP* (since 2015).^51,52^*Audits* have been strongly encouraged since 2013 (e.g. via TARGET) for commissioners to monitor clinicians’ prescribing and guideline compliance.^41^ Such audits prompt practice staff and medicines management teams to identify over-prescribing patterns, discuss issues and identify actions.^31^

The *CMO’s letter* was a social norms feedback intervention in which, from 2014, GPs in high prescribing practices received a letter providing feedback and support to reduce antibiotic prescribing. The letter contained information on the practices’ prescribing compared to other teams and the TARGET ‘treat your infection’ leaflet.^53,54^ The CMO letter was rolled out as an RCT, which showed it successfully helped change antibiotic use in primary care in the short term (<1 year) and when it was repeated in 2017.^25,55^ The letters created a competitive and motivating environment, but concerns were raised that GPs felt they were ‘being told off’ rather than empowered, and that a greater impact was possible from training or structured discussions about prescribing that were also provided.^54^

#### Professional engagement and training (behavioural)

There have been numerous opportunities for healthcare professionals to learn about AMS in undergraduate and postgraduate training, conferences and informal e-learning courses. There is limited evidence surrounding the effectiveness of educational interventions, with most evaluated by surveys after training.

TARGET provided much of this training, for example through FutureLearn e-learning courses with the British Society for Antimicrobial Chemotherapy (BSAC, 2022); train-the-trainer courses (2020) and webinars (2021).^47^ Other training has been developed by NHS England (2021), Royal Pharmaceutical Society (2018), local commissioning organizations and courses linked to interventions.^18^ These opportunities were generally evaluated with post-event feedback, and the implementation in CCGs and NHS Trusts through surveys.^41,56,57^ Some RCTs of the TARGET webinars and strategies supporting primary care prescribers to manage respiratory tract infections have shown them to successfully reduce prescribing.^26,47,58^ However, most studies described self-reported knowledge and do not assess whether training changed behaviour, prescribing or AMR.

Campaigns have also raised awareness amongst professionals. The *Antibiotic Guardian Campaign* was launched by Public Health England in 2014 to gather pledges to improve antibiotic use, with most pledges from healthcare professionals.^59^ It encouraged self-reflection in pharmacists, but reinforced pre-existing beliefs and might only reach individuals already passionate about AMR.^60–62^ There is therefore a risk that such initiatives become ‘echo chambers’ if they do not go beyond already engaged stakeholders, and efforts have been made to ensure campaigns reach a wider range of healthcare professionals.^56^

#### Increasing public awareness (behavioural)

While most interventions have targeted professionals, the public was engaged through the *Antibiotic Guardian Campaign* and *TARGET’s patient-facing resources*. Few studies evaluated awareness-raising initiatives and evidence of effectiveness is weak, relying on surveys with small numbers of respondents or website usage statistics, and not linked to behaviour or prescribing metrics.

The *Keep Antibiotics Working* campaign promoted appropriate antibiotic use in 2017–2019 through TV adverts, posters and leaflets, particularly aimed at high antibiotic users (women aged 20–45 with primary responsibility for family health, and adults over 50).^18,63^ Debates, plays, films and games also engaged the public, including ‘The Mould That Changed the World’ musical since 2020.^64–66^

Similarly to the professional engagement efforts, members of the public pledging to be Antibiotic Guardians have reported higher knowledge of AMR, but this does not necessarily reflect an actual impact as it lacked a baseline knowledge assessment.^62^ International initiatives promoted by England, including *World Antibiotic Awareness Week* and *European Antibiotic Awareness Day* held every November since 2015, have shown only marginal increases in public awareness in England.^67,68^

#### Workforce and governance (structural)

New roles and governance structures have been created to deliver interventions. These have been evaluated locally through surveys assessing what roles exist in hospitals and primary care (e.g. antimicrobial or clinical pharmacists, or senior management teams supporting AMS), but the national governance mechanisms have not been evaluated publicly. There is limited evidence linking workforce changes to prescribing or resistance outcomes.

An independent Scientific Advisory Committee on APRHAI, established in the 2000s consisting of expert clinicians, pharmacists, microbiologists and public health consultants) provides strategic and scientific advice to the government for the NAP, 20-year strategy and developing targets for antibiotic prescribing in primary and secondary care (QP and CQUIN).^69^

Seven *AMS regional leads*, recruited in 2020–2021, lead the coordination of interventions within each region to support NAP delivery alongside regional Infection Prevention Control Leads, the NHS AMR programme and UKHSA.^10^ Pharmacists have had an increased role too, for example community pharmacists in some areas have been critical for increasing access to antibiotics for specific indications such as urinary tract infections through Patient Group Directives, which may increase antibiotic consumption overall but could release pressure on GPs.^70–73^ In hospitals, pharmacists monitored prescribing and worked closely with infectious disease specialists and microbiologists to ensure certain antibiotics (e.g. the AWaRe ‘reserve’ category) require pre-authorization by senior staff members or restrict their use entirely. Eighty percent of AMS programmes in hospitals had multidisciplinary *AMS teams* or *committees* in 2017 but few had financial support, which risks their sustainability.^8,57,71,74^

#### Prescriber tools (technological)

We have classified prescriber tools as interventions used during an interaction between a prescriber and a patient. These tests, technologies and tools are more amenable than other interventions to RCTs to measure their impact on prescribing and clinical outcomes. The evidence base also includes some qualitative process evaluations to understand barriers to implementation.

*Delayed or back-up prescribing* is where a prescription is given but the patient is told to take the antibiotics only if symptoms worsen in a few days. It is a useful stewardship tool that did not increase the risk of complications for patients with respiratory and urinary tract infections.^39,75–77^ Point-of-care *diagnostics tests* were used to detect biomarkers of bacterial infection including CRP and procalcitonin using blood samples, as well as to diagnose urinary tract infections using urine dipstick tests, and respiratory viruses via rapid molecular testing platforms. Such tools may be effective at detecting a bacterial infection, for example an RCT of CRP point-of-care tests saw a 20% absolute reduction in patient-reported antibiotic consumption over four weeks. However, clinicians reported that further support is needed to overcome financial and operational barriers (e.g. physical layout of the practice, consultation duration, extra workload) to embed diagnostics into routine care.^27,78,79^

Furthermore, *computerized decision support tools* (used with electronic health records) have been effective at improving drug selection and decreasing prescribing in certain settings, but clinician engagement can be low.^26,80,81^ Some studies have looked at the synergistic effect of multiple tools, mainly from a behavioural or implementation perspective. One study assessed the effect of multiple initiatives against prescribing by AWaRe category, showing mixed results.^71,82,83^

## Discussion

More than 100 interventions have been officially deployed in England to optimize antimicrobial use since the UK AMR Strategy was published in 2013. This clearly represents significant effort and resource, likely to be underestimated in this review due to additional locally created interventions. Although the number of interventions increased during 2020–2021, COVID-19 has reduced attention to AMS.^84,85^ The interventions aligned to eight categories, although some were delivered as multifaced packages (e.g. guidance documents alongside professional training and local champions). While some interventions were created, delivered and evaluated as if their individual effects could be isolated, in reality most were implemented in parallel, sometimes without reference to one another and often evolved over time. The increase in interventions has probably contributed to the reductions in national antibiotic prescribing since 2014, but it is difficult to attribute success to particular interventions.

### Primary findings: evidence for interventions in England

The increased effort has coincided with a decrease in national antibiotic prescribing rates (although this does not imply causation). Other factors will have affected prescribing during this time, particularly the COVID-19 pandemic that saw reductions in primary care consumption due to fewer GP appointments during national lockdowns while also reducing focus on AMS activities being implemented in the UK.^84^ Nevertheless, the scale and breadth of effort to address antibiotic use across healthcare cannot be understated in England. Interventions have spanned the Health Service, made possible by England’s centralized approach to commissioning and funding, meaning government-led interventions (such as targets) are intended to be aligned with by local organizations.

Structural-level approaches, which are more challenging to implement due to the political backing required and more difficult to evaluate with randomized trials, have demonstrated the largest potential impact on antibiotic prescribing albeit with a small evidence base. One policy and commissioning intervention at the structural level, the QP financial incentive in primary care, reported the largest effect on prescribing. Behavioural level approaches to influence prescribing are relatively easier to implement, thus it is unsurprising to see a proliferation of such approaches in England. There were many guidelines and toolkits, with some effective in hospitals (although relatively few were evaluated). Similarly, interventions that monitor and provide feedback to clinicians in primary care can be effective in the short term. There is, however, a lack of evidence on whether professional engagement and public awareness activities reduce prescribing, mainly as the studies of these interventions focused more on public perceptions or clinician behaviour, rather than prescribing outcomes. Finally, prescriber tools that leverage technological approaches to inform prescribing decisions have higher quality evidence, and reported reductions in prescribing, but can be limited by factors affecting the sustainability of their use such as funding.

Were more than 100 interventions over a decade required to produce a reduction in antimicrobial prescribing to a level that is low enough to slow resistance without causing harm to patients by withholding treatment? The evidence base appears too limited and disjointed to provide a certain answer, but there is a possibility that some specific structural-level interventions have had an effect. Patchiness in the quality of evidence across different intervention types and the lack of connection and comparison of effects between interventions means it is difficult to identify which interventions should be prioritized in the future or replicated in other countries.

### Changing prescribing

There are challenges in defining ‘appropriate’ or ‘sustainable’ antibiotic use; we do not yet know the level at which prescribing would be low enough to reduce the risk of AMR while still ensuring that people are able to access the right antibiotic when they have a bacterial infection requiring drug treatment.^71,86,87^ Nevertheless, several interventions in England have individually been associated with reduced prescribing in the short term, including financial incentives (QP and CQUIN), professional education (TARGET) and social norms approaches (CMO letter), but these interventions were implemented around the same time. Few analyses have investigated the combined or synergistic effects of multiple interventions and some studies will simply be picking up the overall reduction in prescribing during this time. In addition, few studies assessed the sustainability of an intervention over more than a year or its cost effectiveness.

Even when interventions reduced antibiotic prescribing, it is important to determine how this effect was achieved.^88^ Process evaluations or qualitative studies have partially determined this, highlighting that the introduction of national government targets for primary and secondary care with financial incentives helped push AMR up the local agenda, helped local teams prioritize efforts to optimize antibiotic use when there were competing demands and stimulated further activities. For many interventions, having local champions to initiate and lead on AMS was necessary.^89^

Antibiotic use is part of a complex system of numerous partners, simultaneous interventions, heterogenous populations and different stages of the prescribing pathway, making it difficult to draw out the effects of a single intervention from others. Future evaluations should aim to determine how multiple interventions interact, moving the emphasis away from individual programmes. This could be achieved by taking a whole systems approach and following how the intervention interacts with its context, other interventions and system change.^90–92^ AMR could learn from other areas in this respect, such as the evaluation of taxes on sugar in soft drinks in the UK, which triangulated multiple methods and data sources (including expert workshops, interrupted times series analyses and qualitative analysis of media discourses) in order to identify the social, health and economic effects of the levy.^91,93^

### Global context

Globally, there has been a marked increase in interest in AMR since the World Health Assembly resolution in 2015, particularly through the commitment by UN member states to have a NAP in place by 2017 (with many countries now developing their second NAP).^4,22^ With the UN General Assembly High-level Meeting on AMR in 2024, there will be renewed interest in understanding what countries can do to combat AMR through optimizing antibiotic use, for which England can provide a helpful case study given it has reduced prescribing rates over the last decade.

Countries are now faced with the challenge of implementing interventions under the framework of their NAPs. As shown by the vast breadth in interventions in England, a variety of actions targeting prescribing from multiple angles may be needed. WHO’s guidance for NAP development recommends a mix of behavioural, structural and biomedical interventions, but much of the research on AMR globally and in England has focused on individual clinicians’ behaviour rather than creating population-level, system-wide changes to antibiotic prescribing.^13,22,94^ As argued by numerous policy makers and researchers, there is unlikely to be a ‘silver bullet’ intervention that solves AMR.^22^

### Comparison to other studies

A systematic review of studies conducted before 2019 at the global level identified 17 categories of policy interventions. Similar to this current study of England’s interventions, behavioural interventions (guidelines and professional engagement strategies) were most commonly used internationally. Our study did, however, identify more studies of structural changes to the health system than was found globally (e.g. the creation of new workforce and governance structures in the health system). However, it is not possible to say which interventions are likely to have the most impact globally as they have each been evaluated in very particular contexts.^22^

To our knowledge this is the first assessment in the UK covering the range of interventions used to improve antibiotic prescribing. One study comparing strategies used to MRSA infections in Japan and England did identify similar types of intervention while also acknowledging that it was not possible to determine which interventions were responsible for declines in MRSA in England between 2000 and 2017.^21^ A similar study identified that there was also a limited evidence base for behavioural interventions in Canada with few evaluations using rigorous scientific methods.^95^ In Canada and other countries, similarly to what we identified, the impact of individual interventions has been assessed in isolation, rarely considering other interventions implemented at the same time or assessing how interventions operate in synergy or in opposition to one another.

### Strengths and limitations

This is the first review summarizing a comprehensive range of a single country’s national interventions since the first AMR Strategy using both peer-reviewed and grey literature reports. However, it does not cover regional or local interventions, which are less likely to be evaluated in a publicly accessible way, and some other national initiatives used for decades before the 2013–2018 Strategy. It also does not include interventions that indirectly affect antibiotic use, including infection prevention control, vaccination strategies and surveillance. Finally, many of the interventions listed have evolved over time or are no longer implemented, which is often not captured in the literature making it challenging to know how many interventions were ‘active’ at any one point in time.

### Conclusion

Antibiotic use in England has been targeted by numerous interventions implemented in different domains simultaneously, each pulling different levers across the clinical and public health system. High-quality evidence and data on effectiveness of interventions are lacking for most interventions. Interventions using a structural approach had the largest effect on antibiotic use compared to behavioural and technological interventions in England, although other countries may have different experiences. Collectively, the combination of interventions being used may explain the overall decline in prescribing in England but reaching causal conclusions including identifying the most influential interventions is not possible.

## Supplementary Material

dkae061_Supplementary_Data

## Supplementary data

Tables S1 and S3 are available as Supplementary data at *JAC* Online.
