# Supplementary material for: A systematic review of national interventions and policies to optimize antibiotic use in healthcare settings in England
Source: J Antimicrob Chemother. 2024 Mar 20;79(6):1234–47. doi: 10.1093/jac/dkae061 (PMC11144483; doi:10.1093/jac/dkae061)
Supplement: dkae061_Supplementary_Data [file dkae061_supplementary_data.docx]

# Supplementary data

**Table S1: Interventions and their study designs**

| **Type of intervention** | **Study design** | | | | |
| --- | --- | --- | --- | --- | --- |
|  | **Unknown evaluation** | **Qualitative** | **Descriptive and cross sectional** | **Randomised trials** | **Before-after comparison** |
| **STRUCTURAL** | | | | | |
| **Classifications** | - AWaRe Index |  |  |  |  |
| **Policy and commissioning** | - NHS Oversight Framework - NHS Standard Contract - O’Neill review - Hospital action plan to improve stewardship | - NAP^68^ - Quality Premium^11^ | - CQUINs^9,69,70^ - Pharmacy Quality Scheme^16^ - NAP^71^ |  | - Quality Premium^1,8,10,72^ |
| **Workforce & governance** | - Clinical pharmacists in primary care - NHSE Regional AMS leads - Pharmacy Infection professional network (UK Pharmacy Association) - Professional bodies e.g. Royal Colleges - APRHAI - NHS England AMS Regional Leads | - Community pharmacists roles^119^ | - Antimicrobial stewardship committees (in hospitals or primary care)^39^ - Antimicrobial pharmacists ^39,77^ - Community pharmacy antimicrobial stewardship intervention (PAMSI)^58^ - Pharmacist-led stewardship^56^ - Governance structures and processes in hospitals^52^ |  |  |
| **BEHAVIOURAL** | | | | | |
| **Guidance and toolkits** | - AMS Peer review tool - How to...? guides - Intravenous to oral switch - Managing common infections guidance (BNF, NICE, PHE) - Sepsis Action Plan - Specialist Pharmacy Service | - Antimicrobial Self-Assessment Toolkit^73,74^ - ARK-Hospital^2^ - TARGET^23,24^ - Community acquired UTI interventions^67^ - Local common infections guidance^75^ | - Antimicrobial Self-Assessment Toolkit^76^ - NICE guidelines^28^ - Primary care action plan^39^ - Secondary care Action Plan^39,77^ - Start Smart Then Focus ^25,39,77,78^ - TARGET ^16,24–28,39,69,77,79,80^ - Local common infections guidance^75^ | - ARK-Hospital^30^ - TARGET workshop^29^ | - NICE guidelines^77,81^ |
| **Monitoring and feedback** | - Building Rapid Interventions to reduce antibiotic resisTance (BRIT) - Care Quality Commission - NHSE AMS dashboard - NHSE RightCare UTI data packs - Point Prevalence Surveys - PresQIPP - QIPP - Unified Infection database | - CMO letter^36^ | - Auditing prescribing practices^39,77^ - Fingertips data^69,80,82^ - Self-reported tool (SAT)^79^ | - CMO letter^3,83^ - Feedback to clinicians ^84,85^ | - CMO letter^37^ |
| **Professional engagement & training** | - Continuing professional development courses - Health Education England Campaigns - NHSE education and training - RPS AMS training for pharmacists |  | - Antibiotic Guardian Campaign ^6,38^ - Conferences^16,86^ - Fleming Fund’s Commonwealth Partnerships for Antimicrobial Stewardship (CwPAMS)^87^ - GPs trained in integrated medicine^88^ - Primary & secondary care education strategies^39^ - Royal Colleges professional engagement^39^ - Undergraduate education^89^ - TARGET activities, including train the trainer, FutureLearn courses, webinars, campaigns^6,16,77,79^ | - Community pharmacy engagement^40^ - TARGET workshop ^29^ | - Conferences^80^ |
| **Public awareness** |  | - Games^107^ - Peer to peer education^108^ - Patient information^109^ | - Antibiotic Guardian Campaign and youth badge ^6,16,28,43,44,69,80,82,110,111^ - Antibiotic quiz^112^ - Keep antibiotics working campaign^28^ - Patient information^6,109,113^ - World Antibiotic Awareness Week / European Antibiotic Awareness Day^28,80^ - Fear messages^114^ | - Animated films for patients^115^ | - Debates in schools^116^ - Games (e-Bug)^117,118^ - Training educators (e-Bug)^118^ - The Mould that Changed the World theatre production^47^ |
| **TECHNOLOGICAL** | | | | | |
| **Prescriber tools** |  | - Point of Care CRP testing^90–93^ | - Delayed precribing^94,95^ | - Biomarker-guided stewardship^96^ - Clinical prediction scores^97^ - Computerised decision support tools^4^ - Delayed prescribing^98^ - Ear drops (children)^99^ - Probiotics ^100,101^ - Point of Care CRP Testing^5,62^ - Point of Care respiratory virus tests^102^ - Point of Care Urine tests^103^ | - Computerised decision support tool^64^ - Procalcitonin testing^104^ - Point of Care CRP testing^63,105^ - Delayed prescribing & point of care testing^106^ |
| **STUDIES COVERING MULTIPLE CATEGORIES** | | | | | |
| **Studies evaluating multiple types of interventions** |  | - Prescribing behaviour due to multiple interventions^67^ | - AMS education (face-to-face & e-learning), TARGET (patient information, prescriber checklists), Antibiotic Guardian campaign^22^ - Quality Premium, feedback of prescribing data, AMS audits, local incentive schemes, CMO letter, other AMS initiatives^6^ - Hospital structures & processes, including leadership models, AMS teams, policies, rapid testing, authorisation for some antibiotics, reviewing appropriateness, IVOS, automatic alerts feedback, education^52^ |  |  |
| Qualitative studies used interviews and focus groups methods. Descriptive and cross sectional studies were ones conducted at a single point in time, usually through a single survey to gain feedback but also quantitative observational analysis of data (e.g. to track webpage usage). Randomised trials all utilised designs based on RCTs, e.g. cluster randomised trials, pragmatic, open randomised trials, (open adaptive pragmatic parallel group RCT). Before-after comparison used a mix of interrupted time series analyses (often with a controlled or comparative element), or surveys conducted at multiple time points. | | | | | |

**Table S2: Interventions and outcomes evaluated**

| **Type of intervention** | **Focus of evaluation** | | | | | | |
| --- | --- | --- | --- | --- | --- | --- | --- |
|  | **Cost-effectiveness** | **Clinical** | **Microbiological** | **Antibiotic use or prescribing** | **Sustainability** | **Implementation or process** | **Knowledge or**  **behaviour** |
| ***STRUCTURAL*** | | | | | | | |
| **Classifications** |  |  |  |  |  |  |  |
| **Policy and commissioning** |  | - Quality premium^10^ | - Quality premium^1^ | - CQUINs^9,70^ - Quality premium^1,8,10^ | - Quality premium^1,8^ | - CQUINs ^9,69^ - NAP^67,68^ - Pharmacy quality scheme^7,16^ - Quality premium^6,11,72^ |  |
| **Workforce & governance** |  |  |  | - Community pharmacy antimicrobial stewardship intervention (PAMSI) ^57^ - Structures and processes for AMS in hospitals^52^ |  | - Antimicrobial pharmacists ^39,56,77^ - Community pharmacists roles^119^ - Community pharmacy antimicrobial stewardship intervention (PAMSI)^58^ - Antimicrobial stewardship committees (in hospitals or primary care)^39^ - Structures and processes for AMS in hospitals^52^ |  |
| ***BEHAVIOURAL*** | | | | | | | |
| **Guidance and toolkits** |  | - Antibiotic review kit (ARK-hospital) ^30^ - NICE guidelines^81^ |  | - Antibiotic review kit (ARK-hospital) ^30^ - NICE guidelines^77,81^ | - TARGET toolkit^23–27,39,69,79,80,82^ - Start Smart Then Focus^25,39,77^ | - Antimicrobial self-assessment tollkit^74^ - antibiotic review kit (ARK-Hospital)^2^ - Secondary care Action Plan^39,77^ - Local common infections guidance^75^ - Local use of national strategies ^6,39,77^ - Primary care action plan^39^ - Start Smart Then Focus^25,39,77^ - TARGET toolkit^22–27,39,67,69,79,80,82^ | - Start smart then focus ^78^ - TARGET toolkit^26,67^ - NICE guidelines^28,67,77,81^ |
| **Monitoring and feedback** |  |  |  | - CMO letter^3,37,79,83^ - Feedback to clinicians ^84,85^ | - CMO letter ^3,37,79,83^ | - Auditing prescribing practices^6,39,77^ - CMO letter^22,36,67^ - Fingertips data^67,69,80,82^ - Self-reported tool (SAT)^79^ - Benchmarking and feedback of prescribing data^22,52^ | - Fingertips data^67,69,80,82^ - CMO letter^67^ |
| **Professional engagement & training** |  | - Community pharmacy engagement^40^ |  | - GPs trained in integrated medicine^88^ - TARGET workshop ^29^ |  | - Antibiotic Guardian Campaign^67^ - Community pharmacy engagement^40^ - Primary & secondary care education strategies^39^ - TARGET activities, including train the trainer, FutureLearn courses, webinars, campaigns^6,16,77,79^ - Undergraduate education^89^ | - Antibiotic guardian campaign ^38,67^ - Conference^86^ - Fleming Fund’s Commonwealth Partnerships for Antimicrobial Stewardship (CwPAMS)^87^ |
| **Public awareness** |  |  |  | - Patient information^113^ | - Antibiotic guardian campaign^69^ | - Antibiotic quiz^112^ - Games^107^ - Patient information^22,109^ - Peer to peer education^108^ - World Antibiotic Awareness Week / European Antibiotic Awareness Day^28,80^ | - Debates in schools^116^ - Animated films for patients^115^ - Antibiotic guardian campaign^43,44,69^ - Games (e-Bug)^117,118^ - Fear messages^114^ - Keep antibiotics working campaign^28^ - Patient information^109^ - The Mould that Changed the World theatre production^47^ |
| ***TECHNOLOGICAL*** | | | | | | | |
| **Prescriber**  **tools** | - Point of Care CRP Testing^120^ - Point of Care Urine tests^103^ - Outpatient parenteral antimicrobial therapy (OPAT)^121^ | - Computerised decision support tools^4,64,106^ - Delayed prescribing^98^ - Point of Care CRP Testing^120^ - Point of Care respiratory virus tests^102^ | - Biomarker-guided stewardship^96^ - Clinical prediction scores^97^ - Electronic health records to guide AMS^106^ - Point of Care Urine tests^103^ | - Biomarker-guided stewardship^96^ - Computerised decision support tools^4,64,106^ - Delayed prescribing^98^ - Point of Care CRP Testing^5,62,105,120^ - Delayed prescribing & point of care testing combined^66^ - Point of Care respiratory virus tests^102^ - Point of Care Urine tests^103^ - Probiotics ^100,101^ - Procalcitonin testing^104^ | - Computerised decision support tools^4,64^ - Delayed prescribing^98^ - Outpatient parenteral antimicrobial therapy (OPAT)^121^ - Point of Care CRP Testing^120^ | - Computerised decision support tools^4,64,106^ - Clinical algorithm^122^ - Delayed prescribing & point of care testing combined^66^ - Point of Care CRP testing^62,90–93,105^ - FeverPAIN clinical score^67^ - Centor clinical score^67^ | - FeverPAIN clinical score^67^ - Centor clinical score^67^ |
| - *Clinical outcomes include: antibiotic-free days following a procedure, infection incidence, symptom or disease severity (e.g. CURB-65 score for pneumonia, COPD status), symptom or infection duration, length of therapy, length of hospital stay, reattendance to primary care, diagnostic tests results (CRP, prolactin), IVOS, antiviral use, use of X-rays, self-reported referral to GP or pharmacist* - *Microbiological outcomes include: blood cultures sampled before antibiotic initiation, detection of resistant Enterobacterales cultured from stool samples, number of isolates tested against antibiotic during antimicrobial susceptibility testing, resistance to at least one antibiotic,* - *Antibiotic use outcomes include: number of antibiotics prescribed (e.g. per GP practice, per month, per STAR-PU), any antibiotic prescribed (yes/no), defined daily doses (e.g. total, per bed days), patient-reported antibiotic use, duration of antibiotic use, antibiotic days for infections over 1-year, diagnostic accuracy of prediction scores compared to microbiological cultures, achieving antibiotic use targets, percentage of antibiotics prescribed by each AWaRe category* - *Implementation includes: quantitative outcomes (such as adherence to guidelines, use of intervention materials, perceptions of interventions from surveys, AMS-specific job roles present) as well as qualitative studies (e.g. perceptions of interventions from interviews, barriers and facilitators of implementing interventions) and process evaluation* - *Knowledge and behaviour change outcomes include: self-reported change in knowledge, children’s change in knowledge of AMR* | | | | | | | |

**Table S3: Effectiveness of interventions aimed at optimising antibiotic use**

| **Type** | **Study details (intervention name, design, outcomes)** | | **High quality** | **Effect size** |
| --- | --- | --- | --- | --- |
|  | | ***Primary care*** | | |
| Guidance & toolkit | **TARGET workshop, RCT over 32 months in 152 general practices, outcomes: Total ABU (oral antibiotic items dispensed per 1000 patients) & ABU by antibiotic** ^123^ | | **Yes** | **Total ABU reduced 2.7%.**  Amoxicillin/ampicillin reduced 4.4%, Trimethoprim reduced 5.6%, nitrofurantoin no effect |
| Monitoring & feedback | CMO letter, RCT over 6 months in 1909 general practices, outcome: total ABU (antibiotic items prescribed per STAR-PU) and broad spectrum ABU (percentage of broad spectrum items prescribed per STAR-PU)^83^ | | Yes | Total ABU no effect |
|  | **CMO letter, RCT over 5 months in 1581 GP practices (high prescribing practices), outcome: total ABU (antibiotics dispensed per 1000 weighted population, controlling for pas prescribing)**^3^ | | **Yes** | **Total ABU reduced 3.3%** |
|  | **CMO letter, controlled before-after study over 6 months in 7425 GP practices, outcome: total ABU (average STAR-PU-adjusted rate of antibiotic items dispensed)** ^37^ | | **Yes** | **Total ABU reduced 3.69%** |
|  | **Feedback to clinicians, RCT over 12 months in 9 GP practices (RTI patients), outcome: total ABU (rate of antibiotic prescribing for RTI per 1000 patient years over 12 months)**^85^ | | **Yes** | **Total ABU for RTIs reduced 12%** |
|  | Feedback to clinicians, RCT over 5 months in 1401 general practices, outcome: broad spectrum use (proportion of antibiotics which were broad-spectrum)^84^ | | Yes | No effect broad spectrum ABU |
| Policy & commissioning | **Quality premium, before-after study (ITS) over 47 months in primary care, outcome: total ABU (number of antibiotic items prescribed; number of antibiotics prescribed per STAR-PU), broad spectrum ABU (number of broad-spectrum antibiotic items prescribed; and as a percentage of total antibiotics prescribed)**^8^ | | **Yes** | **Total ABU reduced 8.2%** |
|  | **Quality premium, before-after study (ITS) over 72 months in 6882 general practices, outcomes: total ABU (change in rate of antibiotics prescribed per 1000 patients in general practitioner practices)** ^1^ | | **Yes** | **Total ABU reduced 57%** |
| Prescriber tools | Computerised decision support tool, RCT over 12 months in 79 GP practices, outcome: total ABU (antibiotic prescriptions for self-limiting RTIs over 12 months), also adult ABU, children ABU, elderly ABU^4^ | | Yes | **Total ABU reduced 12%** |
|  | Delayed prescribing or clinical scores or antigen tests, RCT over 24 months in 1760 patients with acute sore throat presenting at general practices, outcome: total ABU (patient-reported antibiotic use)^98^ | | Yes | Total ABU 29% lower in clinical score group. Total ABU 27% in antigen test group. Comparator group was delayed prescribing. |
|  | Delayed prescribing & point of care CRP tests, before-after study over 12 months in 9 general practices, outcome total ABU (total antibiotic prescriptions per general practitioner practice)^66^ | | No | Total ABU no effect |
|  | Point of care CRP testing, RCT over 6 months in 8 general practices, outcome patient-reported total ABU (binary outcome of whether antibiotic dispensed or not) ^62^ | | Yes | Total ABU for RTI no effect |
|  | **Point of care CRP testing, RCT over 6 months in 653 participants with COPD (86 general practices), outcome: total ABU (patient reported antibiotic use)**^5^ | | **Yes** | **Total ABU for COPD patients 22% fewer participants in the CRP POCT arm were prescribed antibiotics** |
|  | Point of care urine tests, RCT over 3 months in 329 female patients in primary care, outcome: total ABU (concordant antibiotic use corresponding to laboratory culture results) ^103^ | | Yes | Total ABU in females with UTI no effect |
|  | Probiotics, RCT over 12 months in 310 care home residents, outcome total ABU (cumulative antibiotic administration)^100^ | | Yes | Total ABU no effect |
|  | Probiotics, RCT over 6 months in 1302 asthmatic patients with RTIs in primary care, outcome: total ABU (proportion of patients prescribed antibiotics for RTIs) ^101^ | | Yes | Total ABU asthmatic patients with RTI no effect |
| Professional engagement & training | GP training, observational study over 12 months in 7283 general practices (only 9 with the intervention), outcome: total ABU (antibiotic prescribing rates per STAR-PU, total and for RTIs and UTIs)^88^ | | No | Total ABU reduced 22%. RTI ABU reduced 26%. No effect UTI. |
|  | |  | | |
|  | | ***Secondary care*** | | |
| Guidance & toolkit | **ARK-hospital, RCT over 14 months in 39 hospitals (acute general admission patients), outcome: total ABU (monthly antibiotic consumption as defined daily doses per adult acute general medical admission)** ^30^ | | **Yes** | **Total ABU reduced 4.8% per year** |
|  | NICE guidelines, before-after study over 24 months in 101 hospital patients (paediatrics), outcome: total ABU (proportion of patients prescribed antibiotics) ^81^ | | No | Total ABU in paediatrics reduced 16% |
| Policy & commissioning | CQUINs, Before-after study over 24 months in 116 hospitals, outcome: total ABU (antibiotic consumption in hospital trusts)^9^ | | No | Total ABU no effect.  Carbapenem 8.0% reduction. piperacillin/tazobactam 4.8% reduction |
| Prescriber tools | Biomarker-guided stewardship, RCT over 34 months with 214 patients (in ICU), outcome: total ABU (antibiotic free days over 7 days)^96^ | | Yes | Total ABU no effect |
|  | Computerised decision support tool, before-after study over 48 months in 1 hospital, outcome: total ABU (antibiotic consumption as defined daily doses per 1000 occupied bed-days)^64^ | | No | Total ABU higher without computerised decision support tool. (mean difference − 110.14 defined daily doses/1000 bed day) |
|  | Point of care respiratory virus testing, RCT over 6 months in 720 hospital patients, outcome: total ABU (proportion of patients receiving antibiotic whilst hospitalised)^102^ | | Yes | Total ABU (proportion patients receiving ABU, duration) not changed, over 6 months, hospital |
|  | Procalcitonin testing, controlled before-after study (ITS) over 12 months in 105 hospitals, outcome: total ABU (antibiotic consumption as defined daily doses of antibiotic per admission per week per hospital trust)^104^ | | Yes | Total ABU during COVID-19 no effect |
| Workforce & governance | Structures and processes in hospitals, cross-sectional, outcomes: total ABU and ABU by AWaRe category (antibiotic consumption as defined daily doses per 1000 hospital admission)^52^ | | No | Total ABU no effect |

*Intervention highlighted in bold if it had higher quality evidence & reported reductions in antibiotic use. Higher quality evidence are those which were either RCTs or which utilised an appropriate quasi-experimental design such as interrupted time series analyses.*

*Acronyms: ABU: Antibiotic use. ARK-Hospital: Antibiotic Review Kit-Hospital. AWaRe: Access Watch Reserve. CMO: Chief Medical Officer. CRP: C-Reactive Protein. GP: General Practitioner. ITS: Interrupted Time Series. NICE: National Institute for Health and Care Excellence. RCT: Randomised Controlled Trial. RTI: Respiratory Tract Infection. TARGET: Treat Antibiotics Responsibly, Guidance, Education and Tools.*

**References**

1. Aliabadi S, Anyanwu P, Beech E, *et al.* Effect of antibiotic stewardship interventions in primary care on antimicrobial resistance of Escherichia coli bacteraemia in England (2013–18): a quasi-experimental, ecological, data linkage study. *Lancet Infect Dis* 2021; **0**. Available at: http://www.thelancet.com/article/S1473309921000694/fulltext. Accessed October 7, 2021.

2. Santillo M, Sivyer K, Krusche A, *et al.* Intervention planning for Antibiotic Review Kit (ARK): a digital and behavioural intervention to safely review and reduce antibiotic prescriptions in acute and general medicine. *J Antimicrob Chemother* 2019; **74**: 3362–70. Available at: https://academic.oup.com/jac/article/74/11/3362/5552322. Accessed May 29, 2023.

3. Hallsworth M, Chadborn T, Sallis A, *et al.* Provision of social norm feedback to high prescribers of antibiotics in general practice: a pragmatic national randomised controlled trial. *Lancet* 2016; **387**: 1743–52.

4. Gulliford MC, Juszczyk D, Prevost AT, *et al.* Electronically delivered interventions to reduce antibiotic prescribing for respiratory infections in primary care: cluster RCT using electronic health records and cohort study. *Health Technol Assess* 2019; **23**: 1–72. Available at: https://pubmed.ncbi.nlm.nih.gov/30900550/. Accessed May 14, 2023.

5. Francis NA, Gillespie D, White P, *et al.* C-reactive protein point-of-care testing for safely reducing antibiotics for acute exacerbations of chronic obstructive pulmonary disease: the PACE RCT. *Health Technol Assess* 2020; **24**: 1–108. Available at: https://pubmed.ncbi.nlm.nih.gov/32202490/. Accessed May 29, 2023.

6. Allison R, Lecky DM, Beech E, *et al.* What antimicrobial stewardship strategies do NHS commissioning organizations implement in primary care in England? *JAC Antimicrob Resist* 2020; **2**. Available at: https://academic.oup.com/jacamr/article/2/2/dlaa020/5837000. Accessed May 9, 2022.

7. Hayes C V., Parekh S, Lecky DM, Loader J, Triggs-Hodge C, Ashiru-Oredope D. The National Implementation of a Community Pharmacy Antimicrobial Stewardship Intervention (PAMSI) through the English Pharmacy Quality Scheme 2020 to 2022. *Antibiotics* 2023; **12**: 793. Available at: https://www.mdpi.com/2079-6382/12/4/793. Accessed May 1, 2023.

8. Balinskaite V, Johnson AP, Holmes A, Aylin P. The Impact of a National Antimicrobial Stewardship Program on Antibiotic Prescribing in Primary Care: An Interrupted Time Series Analysis. *Clin Infect Dis* 2019; **69**: 227–32. Available at: https://pubmed.ncbi.nlm.nih.gov/30339190/. Accessed February 4, 2022.

9. Islam J, Ashiru-Oredope D, Budd E, *et al.* A national quality incentive scheme to reduce antibiotic overuse in hospitals: evaluation of perceptions and impact. *J Antimicrob Chemother* 2018; **73**: 1708–13. Available at: https://academic.oup.com/jac/article/73/6/1708/4913756. Accessed May 1, 2023.

10. Balinskaite V, Bou-Antoun S, Johnson AP, Holmes A, Aylin P. An Assessment of Potential Unintended Consequences Following a National Antimicrobial Stewardship Program in England: An Interrupted Time Series Analysis. *Clin Infect Dis* 2019; **69**: 233–42. Available at: https://academic.oup.com/cid/article/69/2/233/5136397. Accessed May 12, 2022.

11. Borek AJ, Anthierens S, Allison R, *et al.* How did a Quality Premium financial incentive influence antibiotic prescribing in primary care? Views of Clinical Commissioning Group and general practice professionals. *J Antimicrob Chemother* 2020; **75**: 2681–8. Available at: https://academic.oup.com/jac/article/75/9/2681/5861493. Accessed May 13, 2022.

12. World Health Organization. *World Health Organization Model List of Essential Medicines, 21st List*. Geneva; 2019.

13. WHO. AWaRe. 2019. Available at: https://adoptaware.org. Accessed November 23, 2020.

14. HM Government. *Tackling antimicrobial resistance 2019–2024 – The UK’s five-year national action plan*. London; 2019.

15. Budd E, Cramp E, Sharland M, *et al.* Adaptation of the WHO Essential Medicines List for national antibiotic stewardship policy in England: being AWaRe. *J Antimicrob Chemother* 2019; **74**: 3384–9. Available at: https://academic.oup.com/jac/article/74/11/3384/5540739. Accessed March 7, 2022.

16. UKHSA. *English surveillance programme for antimicrobial utilisation and resistance (ESPAUR) report 2021 to 2022*. London; 2022.

17. Bowker G, Star S. *Sorting things out: Classification and its consequences*. Cambridge, Massachusetts: The MIT Press; 2000.

18. Cooke J, Llor C, Hopstaken R, Dryden M, Butler C. Respiratory tract infections (RTIs) in primary care: Narrative review of C reactive protein (CRP) point-of-care testing (POCT) and antibacterial use in patients who present with symptoms of RTI. *BMJ Open Respir Res* 2020; **7**.

19. NICE, PHE. Summary of antimicrobial prescribing guidance – managing common infections. 2023.

20. NICE. Antimicrobial stewardship: systems and processes for effective antimicrobial medicine use. 2015.

21. RCGP, Public Health England. TARGET antibiotics toolkit hub. Available at: https://elearning.rcgp.org.uk/course/view.php?id=553. Accessed May 12, 2022.

22. Allison R, Lecky DM, Beech E, *et al.* What Resources Do NHS Commissioning Organisations Use to Support Antimicrobial Stewardship in Primary Care in England? *Antibiotics 2020, Vol 9, Page 158* 2020; **9**: 158. Available at: https://www.mdpi.com/2079-6382/9/4/158/htm. Accessed May 6, 2022.

23. Jones LF, Hawking MKD, Owens R, *et al.* An evaluation of the TARGET (Treat Antibiotics Responsibly; Guidance, Education, Tools) Antibiotics Toolkit to improve antimicrobial stewardship in primary care—is it fit for purpose? *Fam Pract* 2018; **35**: 461–7. Available at: https://academic.oup.com/fampra/article/35/4/461/4780847. Accessed May 20, 2022.

24. Eley C V, Lecky DM, Hayes C V, Mcnulty CA. Is sharing the TARGET respiratory tract infection leaflet feasible in routine general practice to improve patient education and appropriate antibiotic use? A mixed methods study in England with patients and healthcare professionals. *J Infect Prev* 2020: 97–107. Available at: https://doi.org/10.1177/1757177420907698. Accessed May 5, 2023.

25. Ashiru-Oredope D, (ESPAUR) on behalf of the ESP for AU and R, Budd EL, *et al.* Implementation of antimicrobial stewardship interventions recommended by national toolkits in primary and secondary healthcare sectors in England: TARGET and Start Smart Then Focus. *J Antimicrob Chemother* 2016; **71**: 1408–14. Available at: https://academic.oup.com/jac/article/71/5/1408/1751355. Accessed May 5, 2023.

26. Owens R, Jones LF, Moore M, Pilat D, McNulty C. Self-Assessment of Antimicrobial Stewardship in Primary Care: Self-Reported Practice Using the TARGET Primary Care Self-Assessment Tool. *Antibiotics 2017, Vol 6, Page 16* 2017; **6**: 16. Available at: https://www.mdpi.com/2079-6382/6/3/16/htm. Accessed May 5, 2023.

27. Public Health England. *English Surveillance Programme for Antimicrobial Utilisation and Resistance (ESPAUR)*. London; 2018. Available at: https://webarchive.nationalarchives.gov.uk/ukgwa/20181130125613/https://www.gov.uk/government/publications/english-surveillance-programme-antimicrobial-utilisation-and-resistance-espaur-report. Accessed May 12, 2022.

28. UKHSA. *English surveillance programme for antimicrobial utilisation and resistance (ESPAUR) Report 2020 to 2021*. 2021.

29. McNulty C, Hawking M, Lecky D, *et al.* Effects of primary care antimicrobial stewardship outreach on antibiotic use by general practice staff: pragmatic randomized controlled trial of the TARGET antibiotics workshop. *J Antimicrob Chemother* 2018; **73**: 1423–32. Available at: https://academic.oup.com/jac/article/73/5/1423/4909829. Accessed May 12, 2022.

30. Llewelyn MJ, Budgell EP, Laskawiec-Szkonter M, *et al.* Antibiotic review kit for hospitals (ARK-Hospital): a stepped-wedge cluster-randomised controlled trial. *Lancet Infect Dis* 2023; **23**: 207–21. Available at: http://www.thelancet.com/article/S1473309922005084/fulltext. Accessed May 4, 2023.

31. Al-Haboubi M, Trathen A, Black N, Eastmure E, Mays N. Views of health care professionals and policy-makers on the use of surveillance data to combat antimicrobial resistance. *BMC Public Health* 2020; **20**: 1–10. Available at: https://bmcpublichealth.biomedcentral.com/articles/10.1186/s12889-020-8383-8. Accessed May 5, 2023.

32. Cialdini RB, Trost MR. Social influence, social norms, conformity and compliance. In: Gilbert DT, Fiske ST, Lindzey G, eds. *The Handbook of Social Psychology*. New York: McGraw-Hill, 1998; 151–92.

33. PrescQIPP CIC. PrescQIPP . 2018. Available at: https://www.prescqipp.info/. Accessed October 21, 2022.

34. Office for Health Improvement & Disparities. Fingertips. 2022. Available at: https://fingertips.phe.org.uk/. Accessed October 22, 2022.

35. NHS Commissioning Board (North of England Commissioning Support). Chief Medical Officer Letters to High Antibiotic Prescribers. 2017. Available at: https://medicines.necsu.nhs.uk/cmo-letters-to-high-antibiotic-prescribers/. Accessed October 21, 2022.

36. Steels S, Gold N, Palin V, Chadborn T, van Staa TP. Improving Our Understanding and Practice of Antibiotic Prescribing: A Study on the Use of Social Norms Feedback Letters in Primary Care. *Int J Environ Res Public Health* 2021; **18**: 1–10. Available at: /pmc/articles/PMC7967541/. Accessed March 25, 2023.

37. Ratajczak M, Gold N, Hailstone S, Chadborn T. The effectiveness of repeating a social norm feedback intervention to high prescribers of antibiotics in general practice: a national regression discontinuity design. *J Antimicrob Chemother* 2019; **74**: 3603–10. Available at: https://academic.oup.com/jac/article/74/12/3603/5572327. Accessed May 5, 2023.

38. Seaton D, Ashiru-Oredope D, Charlesworth J, Gemmell I, Harrison R. Evaluating UK Pharmacy Workers Knowledge, Attitudes and Behaviour towards Antimicrobial Stewardship and Assessing the Impact of Training in Community Pharmacy. *Pharmacy 2022, Vol 10, Page 98* 2022; **10**: 98. Available at: https://www.mdpi.com/2226-4787/10/4/98/htm. Accessed May 29, 2023.

39. Public Health England. *English surveillance programme for antimicrobial utilisation and resistance (ESPAUR) 2010 to 2014. Report 2015*. London ; 2015. Available at: https://webarchive.nationalarchives.gov.uk/ukgwa/20181130125613/https://www.gov.uk/government/publications/english-surveillance-programme-antimicrobial-utilisation-and-resistance-espaur-report. Accessed May 29, 2023.

40. Ashiru-Oredope D, Doble A, Thornley T, *et al.* Improving Management of Respiratory Tract Infections in Community Pharmacies and Promoting Antimicrobial Stewardship: A Cluster Randomised Control Trial with a Self-Report Behavioural Questionnaire and Process Evaluation. *Pharmacy (Basel)* 2020; **8**: 44. Available at: https://pubmed.ncbi.nlm.nih.gov/32204383/. Accessed May 5, 2023.

41. Public Health England. *Antibiotic Guardian leaflet for health and social care workers/professionals*. 2017. Available at: www.antibioticguardian.com. Accessed October 22, 2022.

42. Kesten JM, Bhattacharya A, Ashiru-Oredope D, Gobin M, Audrey S. The Antibiotic Guardian campaign: A qualitative evaluation of an online pledge-based system focused on making better use of antibiotics. *BMC Public Health* 2017; **18**: 1–13. Available at: https://link.springer.com/articles/10.1186/s12889-017-4552-9. Accessed May 5, 2023.

43. Bhattacharya A, Hopkins S, Sallis A, Budd EL, Ashiru-Oredope D. A process evaluation of the UK-wide Antibiotic Guardian campaign: developing engagement on antimicrobial resistance. *J Public Health (Bangkok)* 2017; **39**: e40–7. Available at: https://academic.oup.com/jpubhealth/article/39/2/e40/3002984. Accessed May 5, 2023.

44. Newitt S, Oloyede O, Puleston R, Hopkins S, Ashiru-Oredope D. Demographic, Knowledge and Impact Analysis of 57,627 Antibiotic Guardians Who Have Pledged to Contribute to Tackling Antimicrobial Resistance. *Antibiotics 2019, Vol 8, Page 21* 2019; **8**: 21. Available at: https://www.mdpi.com/2079-6382/8/1/21/htm. Accessed May 5, 2023.

45. Public Health England. Keep Antibiotics Working . 2019. Available at: https://campaignresources.phe.gov.uk/resources/campaigns/58-keep-antibiotics-working/Overview. Accessed October 21, 2022.

46. The Mould That Changed the World. About. 2022. Available at: https://www.mouldthatchangedtheworld.com/about/. Accessed May 7, 2023.

47. Hall J, Jones L, Robertson G, Hiley R, Nathwani D, Perry MR. ‘The Mould that Changed the World’: Quantitative and qualitative evaluation of children’s knowledge and motivation for behavioural change following participation in an antimicrobial resistance musical. *PLoS One* 2020; **15**. Available at: https://pubmed.ncbi.nlm.nih.gov/33119647/. Accessed May 7, 2023.

48. Ashiru-Oredope D, Lowe T. *Antibiotic guardian: Activities overview September 2014 to September 2016*. 2016. Available at: https://assets.publishing.service.gov.uk/government/uploads/system/uploads/attachment_data/file/570196/activities_overview_antibiotic_guardian_2014_to_2016_.pdf. Accessed May 8, 2023.

49. WHO. World Antimicrobial Awareness Week. 2022. Available at: https://www.who.int/campaigns/world-antimicrobial-awareness-week/2022. Accessed October 22, 2022.

50. Keitoku K, Nishimura Y, Hagiya H, Koyama T, Otsuka F. Impact of the World Antimicrobial Awareness Week on public interest between 2015 and 2020: A Google Trends analysis. *Int J Infect Dis* 2021; **111**: 12–20.

51. UK Government. APRHAI: summary of activities and recommendations, January to December 2019. 2021. Available at: https://www.gov.uk/government/publications/aprhai-summary-of-activities-and-recommendations/aprhai-summary-of-activities-and-recommendations-january-to-december-2019. Accessed May 1, 2023.

52. Scobie A, Budd EL, Harris RJ, Hopkins S, Shetty N. Antimicrobial stewardship: an evaluation of structure and process and their association with antimicrobial prescribing in NHS hospitals in England. *J Antimicrob Chemother* 2019; **74**: 1143–52. Available at: https://academic.oup.com/jac/article/74/4/1143/5288576. Accessed February 4, 2022.

53. NICE. Non-medical prescribing. 20223. Available at: https://bnf.nice.org.uk/medicines-guidance/non-medical-prescribing/. Accessed May 12, 2023.

54. The British Society for Antimicrobial Chemotherapy. Community Pharmacist prescriptions for antibiotics: BSAC highlights need for evaluation of impact on antimicrobial resistance. 2023. Available at: https://bsac.org.uk/community-pharmacist-prescriptions-for-antibiotics-bsac-highlights-need-for-evaluation-of-impact-on-antimicrobial-resistance/. Accessed May 12, 2023.

55. Booth JL, Mullen AB, Thomson DAM, *et al.* Antibiotic treatment of urinary tract infection by community pharmacists: a cross-sectional study. *Br J Gen Pract* 2013; **63**: e244–9. Available at: https://bjgp.org/content/63/609/e244. Accessed June 15, 2023.

56. Wickens HJ, Farrell S, Ashiru-Oredope DAI, Jacklin A, Holmes A. The increasing role of pharmacists in antimicrobial stewardship in English hospitals. *J Antimicrob Chemother* 2013; **68**: 2675–81. Available at: https://pubmed.ncbi.nlm.nih.gov/23825383/. Accessed May 5, 2023.

57. Hayes C V., Lecky DM, Pursey F, *et al.* Mixed-Method Evaluation of a Community Pharmacy Antimicrobial Stewardship Intervention (PAMSI). *Healthcare (Basel)* 2022; **10**. Available at: https://pubmed.ncbi.nlm.nih.gov/35885814/. Accessed May 5, 2023.

58. Allison R, Chapman S, Howard P, *et al.* Feasibility of a community pharmacy antimicrobial stewardship intervention (PAMSI): an innovative approach to improve patients’ understanding of their antibiotics. *JAC Antimicrob Resist* 2020; **2**. Available at: https://academic.oup.com/jacamr/article/2/4/dlaa089/5943021. Accessed May 5, 2023.

59. Little P, Gould C, Williamson I, Moore M, Warner G, Dunleavey J. Pragmatic randomised controlled trial of two prescribing strategies for childhood acute otitis media. *BMJ* 2001; **322**: 336–42. Available at: https://www.bmj.com/content/322/7282/336. Accessed May 14, 2023.

60. Shallcross L, Rockenschaub P, Blackburn R, Nazareth I, Freemantle N, Hayward A. Antibiotic prescribing for lower UTI in elderly patients in primary care and risk of bloodstream infection: A cohort study using electronic health records in England. *PLoS Med* 2020; **17**: e1003336. Available at: https://journals.plos.org/plosmedicine/article?id=10.1371/journal.pmed.1003336. Accessed May 14, 2023.

61. Spurling GKP, Del Mar CB, Dooley L, Foxlee R, Farley R. Delayed antibiotic prescriptions for respiratory infections. *Cochrane Database Syst Rev* 2017; **2017**. Available at: https://www.cochranelibrary.com/cdsr/doi/10.1002/14651858.CD004417.pub5/full. Accessed May 14, 2023.

62. Eley CV, Sharma A, Lee H, Charlett A, Owens R, McNulty CAM. Effects of primary care C-reactive protein point-of-care testing on antibiotic prescribing by general practice staff: Pragmatic randomised controlled trial, England, 2016 and 2017. *Eurosurveillance* 2020; **25**: 1900408. Available at: https://www.eurosurveillance.org/content/10.2807/1560-7917.ES.2020.25.44.1900408. Accessed May 14, 2023.

63. Johnson M, Cross L, Sandison N, Stevenson J, Monks T, Moore M. Funding and policy incentives to encourage implementation of point-of-care C-reactive protein testing for lower respiratory tract infection in NHS primary care: a mixed-methods evaluation. *BMJ Open* 2018; **8**: e024558. Available at: https://bmjopen.bmj.com/content/8/10/e024558. Accessed May 14, 2023.

64. Al Bahar F, Curtis CE, Alhamad HQ. The impact of a computerised decision support system on antibiotic usage in an English hospital. *Int J Clin Pharm* 2020; **42**: 765–71. Available at: https://doi.org/10.1007/s11096-020-01022-3. Accessed May 14, 2023.

65. Rawson TM, Moore LSP, Charani E, *et al.* A systematic review of clinical decision support systems for antimicrobial management: are we failing to investigate these interventions appropriately? *Clin Microbiol Infect* 2017; **23**: 524–32.

66. Tonkin-Crine S, McLeod M, Borek AJ, *et al.* Implementing antibiotic stewardship in high-prescribing English general practices: a mixed-methods study. *Br J Gen Pract* 2023; **73**: e164–75. Available at: https://bjgp.org/content/73/728/e164. Accessed March 24, 2023.

67. Public Health England. *Exploring the implementation of interventions to reduce antibiotic use (ENACT study)*. 2021.

68. Eastmure E, Fraser A, Al-Haboubi M, *et al.* *Evaluation of the Implementation of the UK Antimicrobial Resistance (AMR) Strategy, 2013-2018*. London; 2019. Available at: http://piru.lshtm.ac.uk.

69. Public Health England. *English Surveillance Programme for Antimicrobial Utilisation and Resistance (ESPAUR) Report 2017*. London; 2017. Available at: https://webarchive.nationalarchives.gov.uk/ukgwa/20181130125613/https://www.gov.uk/government/publications/english-surveillance-programme-antimicrobial-utilisation-and-resistance-espaur-report. Accessed May 29, 2023.

70. Powell N, McGraw-Allen K, Menzies A, Peet B, Simmonds C, Wild A. Identifying antibiotic stewardship interventions to meet the NHS England CQUIN: an evaluation of antibiotic ­prescribing against published evidence-based antibiotic audit tools. *Clin Med (Lond)* 2018; **18**: 276. Available at: /pmc/articles/PMC6334038/. Accessed May 29, 2023.

71. Kinoshita T, Tokumasu H, Tanaka S, Kramer A, Kawakami K. Policy implementation for methicillin-resistant Staphylococcus aureus in seven European countries: a comparative analysis from 1999 to 2015. *J Mark Access Health Policy* 2017; **5**: 1351293. Available at: /pmc/articles/PMC5533128/. Accessed May 29, 2023.

72. Anyanwu PE, Pouwels K, Walker A, *et al.* Investigating the mechanism of impact and differential effect of the Quality Premium scheme on antibiotic prescribing in England: a longitudinal study. *BJGP Open* 2020; **4**. Available at: https://bjgpopen.org/content/4/3/bjgpopen20X101052. Accessed October 8, 2021.

73. Bailey C, Tully M, Cooke J. An investigation into the content validity of the Antimicrobial Self-Assessment Toolkit for NHS Trusts (ASAT v15a) using cognitive interviews with antimicrobial pharmacists. *J Clin Pharm Ther* 2015; **40**: 208–12. Available at: https://pubmed.ncbi.nlm.nih.gov/25678341/. Accessed May 29, 2023.

74. Bailey C, Tully M, Cooke J. Perspectives of clinical microbiologists on antimicrobial stewardship programmes within NHS trusts in England. *Antimicrob Resist Infect Control* 2015; **4**: 1–9. Available at: https://aricjournal.biomedcentral.com/articles/10.1186/s13756-015-0090-3. Accessed May 29, 2023.

75. Allison R, Lecky DM, Beech E, *et al.* Local implementation of national guidance on management of common infections in primary care in England. *Pharmaceutical Journal* 2020.

76. Bailey C, Tully MP, Pampaka M, Cooke J. Rasch analysis of the Antimicrobial Self-Assessment Toolkit for National Health Service (NHS) Trusts (ASAT v17). *J Antimicrob Chemother* 2017; **72**: 604–13. Available at: https://pubmed.ncbi.nlm.nih.gov/27798214/. Accessed May 29, 2023.

77. Public Health England. *English surveillance programme for antimicrobial utilisation and resistance (ESPAUR) Report 2014*. London; 2014. Available at: https://webarchive.nationalarchives.gov.uk/ukgwa/20181130125613/https://www.gov.uk/government/publications/english-surveillance-programme-antimicrobial-utilisation-and-resistance-espaur-report. Accessed May 29, 2023.

78. Roope LSJ, Buchanan J, Morrell L, *et al.* Why do hospital prescribers continue antibiotics when it is safe to stop? Results of a choice experiment survey. *BMC Med* 2020; **18**: 1–11. Available at: https://bmcmedicine.biomedcentral.com/articles/10.1186/s12916-020-01660-4. Accessed May 29, 2023.

79. Public Health England. *English surveillance programme for antimicrobial utilisation and resistance (ESPAUR) report 2019 to 2020*. London; 2020. Available at: https://webarchive.nationalarchives.gov.uk/ukgwa/20211022024510/https://www.gov.uk/government/publications/english-surveillance-programme-antimicrobial-utilisation-and-resistance-espaur-report. Accessed May 29, 2023.

80. Public Health England. *English surveillance programme for antimicrobial utilisation and resistance (ESPAUR) report 2018-2019*. London; 2019. Available at: https://webarchive.nationalarchives.gov.uk/ukgwa/20200806045257/https://www.gov.uk/government/publications/english-surveillance-programme-antimicrobial-utilisation-and-resistance-espaur-report. Accessed May 29, 2023.

81. Breakell R, Thorndyke B, Clennett J, Harkensee C. Reducing unnecessary chest X-rays, antibiotics and bronchodilators through implementation of the NICE bronchiolitis guideline. *Eur J Pediatr* 2018; **177**: 47–51. Available at: https://link.springer.com/article/10.1007/s00431-017-3034-5. Accessed May 5, 2023.

82. Public Health England. *English surveillance programme for antimicrobial utilisation and resistance (ESPAUR) Report 2016*. London; 2016. Available at: https://webarchive.nationalarchives.gov.uk/ukgwa/20181130125613/https://www.gov.uk/government/publications/english-surveillance-programme-antimicrobial-utilisation-and-resistance-espaur-report. Accessed May 29, 2023.

83. Gold N, Sallis A, Saei A, *et al.* Using text and charts to provide social norm feedback to general practices with high overall and high broad-spectrum antibiotic prescribing: a series of national randomised controlled trials. *Trials* 2022; **23**: 1–15. Available at: https://trialsjournal.biomedcentral.com/articles/10.1186/s13063-022-06373-y. Accessed May 29, 2023.

84. Curtis HJ, Bacon S, Croker R, *et al.* Evaluating the impact of a very low-cost intervention to increase practices’ engagement with data and change prescribing behaviour: a randomized trial in English primary care. *Fam Pract* 2021; **38**: 373–80. Available at: https://academic.oup.com/fampra/article/38/4/373/6203915. Accessed May 29, 2023.

85. Gulliford MC, Prevost AT, Charlton J, *et al.* Effectiveness and safety of electronically delivered prescribing feedback and decision support on antibiotic use for respiratory illness in primary care: REDUCE cluster randomised trial. *BMJ* 2019; **364**: 236. Available at: https://www.bmj.com/content/364/bmj.l236. Accessed May 29, 2023.

86. Guilding C, Hardisty J, Randles E, *et al.* Designing and evaluating an interprofessional education conference approach to antimicrobial education. *BMC Med Educ* 2020; **20**: 1–13. Available at: https://bmcmededuc.biomedcentral.com/articles/10.1186/s12909-020-02252-9. Accessed May 29, 2023.

87. Brandish C, Garraghan F, Ng BY, Russell-Hobbs K, Olaoye O, Ashiru-Oredope D. Assessing the impact of a global health fellowship on pharmacists’ leadership skills and consideration of benefits to the national health service (NHS) in the United Kingdom. *Healthcare (Switzerland)* 2021; **9**: 890. Available at: https://www.mdpi.com/2227-9032/9/7/890/htm. Accessed May 29, 2023.

88. Van Der Werf ET, Duncan LJ, Von Flotow P, Baars EW. Do NHS GP surgeries employing GPs additionally trained in integrative or complementary medicine have lower antibiotic prescribing rates? Retrospective cross-sectional analysis of national primary care prescribing data in England in 2016. *BMJ Open* 2018; **8**: e020488. Available at: https://bmjopen.bmj.com/content/8/3/e020488. Accessed May 29, 2023.

89. Castro-Sánchez E, Drumright LN, Gharbi M, Farrell S, Holmes AH. Mapping Antimicrobial Stewardship in Undergraduate Medical, Dental, Pharmacy, Nursing and Veterinary Education in the United Kingdom. *PLoS One* 2016; **11**: e0150056. Available at: https://journals.plos.org/plosone/article?id=10.1371/journal.pone.0150056. Accessed May 29, 2023.

90. Eley CV, Sharma A, Lecky DM, Lee H, McNulty CAM. Qualitative study to explore the views of general practice staff on the use of point-of-care C reactive protein testing for the management of lower respiratory tract infections in routine general practice in England. *BMJ Open* 2018; **8**: e023925. Available at: https://bmjopen.bmj.com/content/8/10/e023925. Accessed May 14, 2023.

91. Phillips R, Stanton H, Gillespie D, *et al.* C-reactive protein-guided antibiotic prescribing for COPD exacerbations: a qualitative evaluation. *Br J Gen Pract* 2020; **70**: E505–13. Available at: https://pubmed.ncbi.nlm.nih.gov/32424045/. Accessed May 14, 2023.

92. Hughes A, Gwyn L, Harris S, Clarke C. Evaluating a point-of-care C-reactive protein test to support antibiotic prescribing decisions in a general practice - The Pharmaceutical Journal. *Pharmaceutical Journal* 2016. Available at: https://pharmaceutical-journal.com/article/research/evaluating-a-point-of-care-c-reactive-protein-test-to-support-antibiotic-prescribing-decisions-in-a-general-practice. Accessed May 29, 2023.

93. Borek AJ, Campbell A, Dent E, *et al.* Implementing interventions to reduce antibiotic use: a qualitative study in high-prescribing practices. *BMC Fam Pract* 2021; **22**: 1–11. Available at: https://bmcprimcare.biomedcentral.com/articles/10.1186/s12875-021-01371-6. Accessed May 29, 2023.

94. Morrell L, Buchanan J, Roope LSJ, *et al.* Delayed Antibiotic Prescription by General Practitioners in the UK: A Stated-Choice Study. *Antibiotics* 2020; **9**: 1–19. Available at: https://www.ncbi.nlm.nih.gov/pmc/articles/PMC7558347/. Accessed May 14, 2023.

95. Morrell L, Buchanan J, Roope LSJ, *et al.* Public preferences for delayed or immediate antibiotic prescriptions in UK primary care: A choice experiment. *PLoS Med* 2021; **18**: e1003737. Available at: https://journals.plos.org/plosmedicine/article?id=10.1371/journal.pmed.1003737. Accessed May 14, 2023.

96. Hellyer TP, McAuley DF, Walsh TS, *et al.* Biomarker-guided antibiotic stewardship in suspected ventilator-associated pneumonia (VAPrapid2): a randomised controlled trial and process evaluation. *Lancet Respir Med* 2020; **8**: 182–91. Available at: http://www.thelancet.com/article/S2213260019303674/fulltext. Accessed May 29, 2023.

97. Seeley A, Fanshawe T, Voysey M, Hay A, Moore M, Hayward G. Diagnostic accuracy of Fever-PAIN and Centor criteria for bacterial throat infection in adults with sore throat: a secondary analysis of a randomised controlled trial. *BJGP Open* 2021; **5**. Available at: https://bjgpopen.org/content/5/6/BJGPO.2021.0122. Accessed June 10, 2023.

98. Little P, Richard Hobbs FD, Moore M, *et al.* Clinical score and rapid antigen detection test to guide antibiotic use for sore throats: randomised controlled trial of PRISM (primary care streptococcal management). *BMJ* 2013; **347**. Available at: https://www.bmj.com/content/347/bmj.f5806. Accessed May 14, 2023.

99. Hay AD, Downing H, Francis NA, *et al.* Anaesthetic-analgesic ear drops to reduce antibiotic consumption in children with acute otitis media: the CEDAR RCT. *Health Technol Assess* 2019; **23**: 1. Available at: https://www.ncbi.nlm.nih.gov/pmc/articles/PMC6661537/. Accessed May 29, 2023.

100. Butler CC, Lau M, Gillespie D, *et al.* Effect of Probiotic Use on Antibiotic Administration Among Care Home Residents: A Randomized Clinical Trial. *JAMA* 2020; **324**: 47–56. Available at: https://jamanetwork.com/journals/jama/fullarticle/2767862. Accessed May 29, 2023.

101. Smith TDH, Watt H, Gunn L, Car J, Boyle RJ. Recommending Oral Probiotics to Reduce Winter Antibiotic Prescriptions in People With Asthma: A Pragmatic Randomized Controlled Trial. *Ann Fam Med* 2016; **14**: 422–30. Available at: https://www.annfammed.org/content/14/5/422. Accessed May 29, 2023.

102. Brendish NJ, Malachira AK, Armstrong L, *et al.* Routine molecular point-of-care testing for respiratory viruses in adults presenting to hospital with acute respiratory illness (ResPOC): a pragmatic, open-label, randomised controlled trial. *Lancet Respir Med* 2017; **5**: 401–11. Available at: https://pubmed.ncbi.nlm.nih.gov/28392237/. Accessed May 14, 2023.

103. Butler CC, Francis NA, Thomas-Jones E, *et al.* Point-of-care urine culture for managing urinary tract infection in primary care: a randomised controlled trial of clinical and cost-effectiveness. *Br J Gen Pract* 2018; **68**: e268–78. Available at: https://pubmed.ncbi.nlm.nih.gov/29483078/. Accessed May 14, 2023.

104. Llewelyn MJ, Grozeva D, Howard P, *et al.* Impact of introducing procalcitonin testing on antibiotic usage in acute NHS hospitals during the first wave of COVID-19 in the UK: a controlled interrupted time series analysis of organization-level data. *J Antimicrob Chemother* 2022; **77**: 1189–96. Available at: https://pubmed.ncbi.nlm.nih.gov/35137110/. Accessed May 14, 2023.

105. Dixon S, Fanshawe TR, Mwandigha L, *et al.* The Impact of Point-of-Care Blood C-Reactive Protein Testing on Prescribing Antibiotics in Out-of-Hours Primary Care: A Mixed Methods Evaluation. *Antibiotics (Basel)* 2022; **11**. Available at: https://pubmed.ncbi.nlm.nih.gov/35892398/. Accessed May 14, 2023.

106. Dutey-Magni PF, Gill MJ, McNulty D, *et al.* Feasibility study of hospital antimicrobial stewardship analytics using electronic health records. *JAC Antimicrob Resist* 2021; **3**. Available at: https://www.ncbi.nlm.nih.gov/pmc/articles/PMC8210026/. Accessed June 10, 2023.

107. Castro-Sánchez E, Sood A, Rawson TM, Firth J, Holmes AH. Forecasting Implementation, Adoption, and Evaluation Challenges for an Electronic Game–Based Antimicrobial Stewardship Intervention: Co-Design Workshop With Multidisciplinary Stakeholders. *J Med Internet Res* 2019; **21**. Available at: /pmc/articles/PMC6746106/. Accessed May 29, 2023.

108. McNulty CAM, Brown CL, Syeda RB, *et al.* Teacher and Student Views on the Feasibility of Peer to Peer Education as a Model to Educate 16–18 Year Olds on Prudent Antibiotic Use—A Qualitative Study. *Antibiotics 2020, Vol 9, Page 194* 2020; **9**: 194. Available at: https://www.mdpi.com/2079-6382/9/4/194/htm. Accessed May 29, 2023.

109. Van Hecke O, Lee JJ, Butler CC, Moore M, Tonkin-Crine S. Using evidence-based infographics to increase parents’ understanding about antibiotic use and antibiotic resistance: a proof-of-concept study. *JAC Antimicrob Resist* 2020; **2**. Available at: https://www.ncbi.nlm.nih.gov/pmc/articles/PMC8210337/. Accessed May 29, 2023.

110. Chaintarli K, Ingle SM, Bhattacharya A, Ashiru-Oredope D, Oliver I, Gobin M. Impact of a United Kingdom-wide campaign to tackle antimicrobial resistance on self-reported knowledge and behaviour change. *BMC Public Health* 2016; **16**: 1–9. Available at: https://bmcpublichealth.biomedcentral.com/articles/10.1186/s12889-016-3057-2. Accessed May 8, 2023.

111. Hayes C V., Eley C V., Ashiru-Oredope D, Hann M, McNulty CAM. Development and pilot evaluation of an educational programme on infection prevention and antibiotics with English and Scottish youth groups, informed by COM-B. *J Infect Prev* 2021; **22**: 212–9. Available at: https://journals.sagepub.com/doi/full/10.1177/17571774211012463. Accessed May 29, 2023.

112. Langford BJ, Laguio-Vila MR. 190. Which antibiotic are you? Evaluation of a global antibiotic awareness personality quiz. *Open Forum Infect Dis* 2020; **7**: S101–S101. Available at: https://academic.oup.com/ofid/article/7/Supplement_1/S101/6058514. Accessed May 29, 2023.

113. Thorpe A, Sirota M, Orbell S, Juanchich M. Effect of information on reducing inappropriate expectations and requests for antibiotics. *Br J Psychol* 2021; **112**: 804–27. Available at: https://pubmed.ncbi.nlm.nih.gov/33543779/. Accessed May 29, 2023.

114. Roope LSJ, Tonkin-Crine S, Herd N, *et al.* Reducing expectations for antibiotics in primary care: A randomised experiment to test the response to fear-based messages about antimicrobial resistance. *BMC Med* 2020; **18**: 1–11. Available at: https://bmcmedicine.biomedcentral.com/articles/10.1186/s12916-020-01553-6. Accessed May 29, 2023.

115. Wilding S, Kettu V, Thompson W, *et al.* Development and randomized controlled trial of an animated film aimed at reducing behaviours for acquiring antibiotics. *JAC Antimicrob Resist* 2021; **3**. Available at: https://academic.oup.com/jacamr/article/3/2/dlab083/6301451. Accessed May 8, 2023.

116. Young VL, Berry M, Verlander NQ, Ridgway A, McNulty CAM. Using debate to educate young people in schools about antibiotic use and resistance: A before and after evaluation using a questionnaire survey. *J Infect Prev* 2019; **20**: 281–8. Available at: https://journals.sagepub.com/doi/10.1177/1757177419862039. Accessed May 29, 2023.

117. Eley CV, Young VL, Hayes CV, Verlander NQ, McNulty CAM. Young People’s Knowledge of Antibiotics and Vaccinations and Increasing This Knowledge Through Gaming: Mixed-Methods Study Using e-Bug. *JMIR Serious Games* 2019; **7**. Available at: https://www.ncbi.nlm.nih.gov/pmc/articles/PMC6376338/. Accessed May 29, 2023.

118. Hayes C, Eley C, Brown C, *et al.* Improving educator’s knowledge and confidence to teach infection prevention and antimicrobial resistance. *Health Educ J* 2021; **80**: 131–44. Available at: https://journals.sagepub.com/doi/10.1177/0017896920949597. Accessed May 29, 2023.

119. Jones LF, Owens R, Sallis A, *et al.* Qualitative study using interviews and focus groups to explore the current and potential for antimicrobial stewardship in community pharmacy informed by the Theoretical Domains Framework. *BMJ Open* 2018; **8**. Available at: https://pubmed.ncbi.nlm.nih.gov/30593557/. Accessed May 29, 2023.

120. Hunter R. Cost-Effectiveness of Point-of-Care C-Reactive Protein Tests for Respiratory Tract Infection in Primary Care in England. *Adv Ther* 2015; **32**: 69–85. Available at: https://link.springer.com/article/10.1007/s12325-015-0180-x. Accessed June 10, 2023.

121. Dimitrova M, Gilchrist M, Seaton RA. Outpatient parenteral antimicrobial therapy (OPAT) versus inpatient care in the UK: a health economic assessment for six key diagnoses. *BMJ Open* 2021; **11**. Available at: https://pubmed.ncbi.nlm.nih.gov/34588251/. Accessed June 10, 2023.

122. Potter R, Campbell A, Ellard DR, *et al.* Multifaceted intervention to Reduce Antimicrobial Prescribing in Care Homes: a process evaluation of a UK-based non-randomised feasibility study. *BMJ Open* 2019; **9**: e032185. Available at: https://bmjopen.bmj.com/content/9/11/e032185. Accessed April 14, 2023.

123. McNulty C, Hawking M, Lecky D, *et al.* Effects of primary care antimicrobial stewardship outreach on antibiotic use by general practice staff: pragmatic randomized controlled trial of the TARGET antibiotics workshop. *J Antimicrob Chemother* 2018; **73**: 1423. Available at: /pmc/articles/PMC5909634/. Accessed May 4, 2023.
